# Glutamine increases stability of TPH1 mRNA via p38 mitogen-activated kinase in mouse mastocytoma cells

**DOI:** 10.1007/s11033-022-07693-7

**Published:** 2022-11-04

**Authors:** Heeyoung Park, Chang-Wook Lee, Jieun Kang, Ali Sadra, Sung-Oh Huh

**Affiliations:** Department of Pharmacology, College of Medicine, Institute of Natural Medicine, Hallym University, Chuncheon 24252, Gangwon-Do, South Korea

**Keywords:** Tryptophan hydroxylase 1, Glutamine, p38 MAP kinase, mRNA stability Heeyoung Park and Chang-Wook Lee contributed equally as the first author.

## Abstract

Expression changes for tryptophan hydroxylase 1 (TPH1), the rate-limiting enzyme in serotonin synthesis, by environmental glutamine (GLN) were examined in mouse mastocytoma-derived P815-HTR cells. GLN-treated cells exhibited a robust increase in TPH1 mRNA after a 6 h exposure to GLN. 6-Diazo-5-oxo-L-norleucine (DON), a glutamine-utilizing glutaminase inhibitor, significantly inhibited the GLN-induction of TPH1 mRNA. Nuclear run-on assays and mRNA decay experiments demonstrated that the primary mechanism leading to increased TPH1 mRNA levels was not due to transcriptional changes, but rather due to increased TPH1 RNA stability induced by GLN. Treatment with GLN also led to activation of p38 MAP kinase, but not p42/44 MAPK. In addition, SB203580, a p38 MAP kinase specific inhibitor, completely abolished the GLN-mediated increase of TPH1 mRNA levels, suggesting the pathway stabilizing TPH1 mRNA might be mediated by the activated p38 MAP kinase pathway. Additionally, SB203580 significantly reduced the stability of TPH1 mRNA, and this reduction of the stability was not affected by GLN in the culture medium, implying a sequential signaling from GLN being mediated by p38 MAP kinase, resulting in alteration of TPH1 mRNA stability. TPH1 mRNA stability loss was also dependent on de novo protein synthesis as shown by treatment of cells with a transcriptional/translational blocker. We provide evidence that TPH1 mRNA levels are increased in response to increased exogenous GLN in mouse mastocytoma cells via a stabilization of TPH1 mRNA due to the activity of the p38 MAP kinase.

**Supplementary Information:**

The online version contains supplementary material available at 10.1007/s11033-022-07693-7.

## Introduction

As a neurotransmitter in the CNS, serotonin or 5-hydroxytryptamine (5-HT), has roles in a number of behavioral disorders such as depression, substance abuse, schizophrenia and autism [1]. In the CNS, the primary source of serotonin are the serotonergic neurons. However, the majority of serotonin is outside of the CNS, with the enterochromaffin (EC) cells of gastrointestinal epithelial tract making about 90% of serotonin in the body [2]. The roles of serotonin in the periphery are quite diverse, such as being involved in gastrointestinal functions, production of insulin, cell bioenergetics, bone homeostasis, and blood coagulation. Serotonin has also been detected as being covalently integrated into proteins in a process called serotonylation [3]. Many substrates of serotonylation have been identified; these include extracellular matrix components such as fibronectin and certain GTPase proteins. The discovered serotonylation dependent activities are numerous including contraction of smooth muscle and activation of platelets [4, 5]. Serotonin is the precursor to N-acetylserotonin (NAS) and melatonin, both with their own biological functions. Serotonin and NAS are also detectable in serum, and these serum supplies are conjectured to serve as substrates for production of melatonin [6–8].

The rate-limiting step in serotonin synthesis is catalyzed by tryptophan hydroxylase (TPH), with TPH activity detected in serotonergic neurons in the CNS, pineal gland, retina, gut intestinal and pancreatic EC cells [9]. TPH activity is also detected in skin keratinocytes, fibroblasts and melanocytes [10]. In mammals, there are two distinct genes with tryptophan hydroxylase activity, TPH1 and TPH2. TPH1 is expressed in most of the cell types mentioned above for TPH activity; on the other hand, TPH2 is almost entirely present in the brain and the neuronal cells with two alternative splice variants described for it [11–13]. TPH2 is also detected in human retinal pigment epithelium cells as part of the melatoninergic system [14].

Little is known about the regulation of TPH1 gene expression, although a few transcriptional mechanisms for TPH1 have been described [15–18]. Difficulties in the study of TPH1 have concerned the relatively low levels of TPH1 in various tissues and its inherent instability. For P815-HTR cells, a transformed mouse mastocytoma cell line, there are reasonable levels of TPH1, allowing a study of TPH1 message regulation and the cells being used as a source of TPH1 [19]. TPH1 is the predominant form of the enzyme expressed in P815-HTR cells, having a molecular weight of 51 kDa [20].

Environmental glutamine (GLN) is a major metabolite, known to affect a myriad of pathways in the cell as the result of its role in cell metabolism [21], and a role for exogenous GLN in regulation of serotonin synthesis via regulation of TPH1 levels was sought. Although GLN can be manufactured by the cell, the majority of cells require exogenous GLN as it is also the most abundant amino acid in the bloodstream [21]. In cultured cells, for example, millimolar levels of GLN in the media are required for survival. In the body, circulating GLN is supplied mostly by the liver, lung, adipocytes and skeletal muscle. These cells produce GLN by various means of cellular synthesis and breakdown of protein, and their contributions to the levels of circulating GLN are modified in various physiological states such as feeding and starvation [22]. Thus, environmental GLN may play a key physiological role in regulating serotonin levels via TPH1.

We demonstrate that TPH1 mRNA is augmented upon exposure to exogenous GLN in mouse mastocytoma cells; this was via stabilization of TPH1 mRNA. Regulation of mRNA turnover such as with RNA stability is important in regulation of gene expression in various organisms for issues where the levels of a given mRNA can dramatically change due to its instability [23] and various cellular processes are regulated by changes in mRNA half-life [24]. We focused on the regulation of TPH1 mRNA as to recover the details of expression control from gene transcription, followed by an assay of RNA stability. We also investigated the effects of p38 MAPK activity on TPH1 mRNA levels in the P815-HTR cells in presence of exogenous GLN, as GLN metabolism has been shown to stimulate intestinal cell MAPKs [25]. For p38 MAPK, it has been shown to be induced by GLN/ glycine isosmotic cell swelling [26]. We show that for TPH1, the p38 MAPK stabilizes TPH1 mRNA and presents a major pathway in regulation of TPH1 levels.

## Materials and methods

### Cell culture

P815-HTR mouse mastocytoma line was obtained from ATCC. Cell culture media and chemicals were purchased from Sigma-Aldrich, unless otherwise specified. The P815-HTR cells was grown in 100-mm culture dishes at 37 °C under a humidified atmosphere of 5% CO_2_ and 95% air. The cells were maintained under culture conditions in Dulbecco’s modified Eagle’s medium (DMEM) supplemented with 10% fetal bovine serum, 20 µg/ml gentamicin and with or without added 4 mM L-glutamine (GLN).

### Northern blotting

For changes in the levels of TPH1 RNA message, the cells were incubated with either 6-diazo-5-oxo-L-norleucine (DON) (glutamine antagonist), SB203580 (p38 MAPK inhibitor), PD98059 (p42/44 MAPK inhibitor) or wortmannin (PI3K inhibitor) at the indicated concentrations in the figure legends for 6 h with control media. Total RNA was extracted and the RNA (5 µg) was separated by 1% agarose/formaldehyde gel electrophoresis and transferred to a Hybond-N + nylon membrane (Amersham Life Sciences, UK). Digoxigenine (DIG)-labeled RNA probes for Northern blot analysis were synthesized with a DIG RNA Labeling Kit (Boehringer Mannheim). Hybridization of the labeled probes and conjugation with an anti-DIG antibody-alkaline phosphatase complex were carried out with a DIG Nucleic Acid Detection Kit (Boehringer Mannheim) according to the manufacturer. The phosphatase complex on the nylon membrane was visualized with the chemiluminescent substrate CSPD (Boehringer Mannheim) according to the manufacturer’s recommendations.

### Western blotting

P815-HTR cells were lysed in radio-immunoprecipitation assay buffer (RIPA) buffer (50 mM Tris-HCl, pH 7.5, 150 mM NaCl, 1 mM EGTA, 1 mM EDTA, 1% Triton X-100, 1 mM Na_3_VO_4_, 5 mM NaF, and a protease inhibitor cocktail). A total of 30 µg of protein was separated per lane on 10% SDS-polyacrylamide gels, followed by transfer onto PVDF membranes (Millipore). The membranes were blocked in TBST (Tris-buffered saline containing 0.1% Tween-20) supplemented with 5% nonfat milk. Probing with the primary antibody was overnight at 4 °C; the blot was washed three times with TBST, and then incubated with appropriate secondary antibody (anti-rabbit HRP). After washing the blots three times in TBST, the protein bands were detected using a Western HRP substrate ECL kit (Luminata Forte, Millipore) and chemiluminescence imaging (Fusion FX, Vilber Lourmat). Primary antibodies were rabbit anti-phospho-p38 MAPK (Thr180/Tyr182) (Cell Signaling #9211; 1: 1000), rabbit anti-p38 MAPK (Cell Signaling #9212; 1: 1000), rabbit anti-phospho-Erk1/2 (Thr202/Tyr204) (Cell signaling #9101; 1: 1000), and rabbit anti-Erk1/2 (Cell Signaling #9102; 1: 1000). Secondary antibody was goat anti-rabbit IgG HRP (Thermo Fisher; 1: 5000).

### Nuclear run-on assay

Nuclear run-on assay was performed to determine transcription initiation rate. The assay was performed as described by Greenberg and Bender and using a modification of published methods [27]. All procedures involving extraction of the nuclei were performed at 4 °C. For in vitro transcription reactions, 3 ⋅ 10 ^7^ nuclei were incubated for 30 min at 30 °C in transcription buffer (Greenberg. M) containing 250 mCi of [α-^32^P] UTP. Following chromatin disruption with the high salt buffer, DNase treatment, and isopropyl alcohol precipitation, RNA was resuspended in 0.1% SDS, diluted with lysis buffer RTL (Qiagen), and then purified using the RNeasy total RNA extraction kit (Qiagen) and following the manufacturer’s instructions. Transcripts were captured by linearized and denatured plasmids (described above for Northern blotting) were immobilized on nylon membranes at 5 µg/slot. Capture membranes were blocked for 15–30 min in prehybridization solution (Greenberg, M.) at 65 °C and then incubated with 1 ⋅ 10 ^7^ cpm/ml of purified in vitro labeled RNA diluted in hybridization solution (Greenberg, M.) for 30 h at 65 °C. After extensive washing at 65 °C with 1⋅ SSC containing 0.1% SDS, the membranes were treated with 50 µg/ml RNase A (Qiagen) for 30 min at room temperature and then washed at room temperature with 1⋅ SSC containing 0.1% SDS. Captured transcripts were detected by exposing the membranes to x-ray film (Reflection, NEN Life Science).

### RNA stability assay

P815-HTR cells were seeded in 6-well plates in complete DMEM for 12 h. The cells were then rinsed twice with GLN-free medium, fed with GLN-free media supplemented with or without 4 mM GLN in combinations of 10 µM SB203580 (Tocris), 10 µg/ml actinomycin D (Sigma) or 100 µM 5,6-dichloro-1-b-D-ribofuranosylbenzimidazole (DRB, Calbiochem) in. The SB203580 reagent was added from a 10 mM stock in DMSO, and actinomycin D was added from a 10 mg/ml stock in absolute ethanol. The DRB was added from a 100 mM stock in DMSO. The control cultures were treated with the given carrier solution without the added drug. At the indicated times after treatment, the cultures were harvested and total RNA was isolated as described for Northern blotting.

### Statistical analysis

The experiments were performed at least 3 times. Statistical analyses were via GraphPad Prism version 5 software (GraphPad). The data are presented as mean ± standard error, and were compared statistically by Student-t test (for 2 groups) and ANOVA with “Tukey’s Multiple Comparison Test” (for more than 2 groups). Significant differences were indicated for p < 0.05.

## Results

### Levels of TPH1 mRNA in P815-HTR cells were increased by presence of GLN, which was blocked by a GLN antagonist

**Fig. 1:**
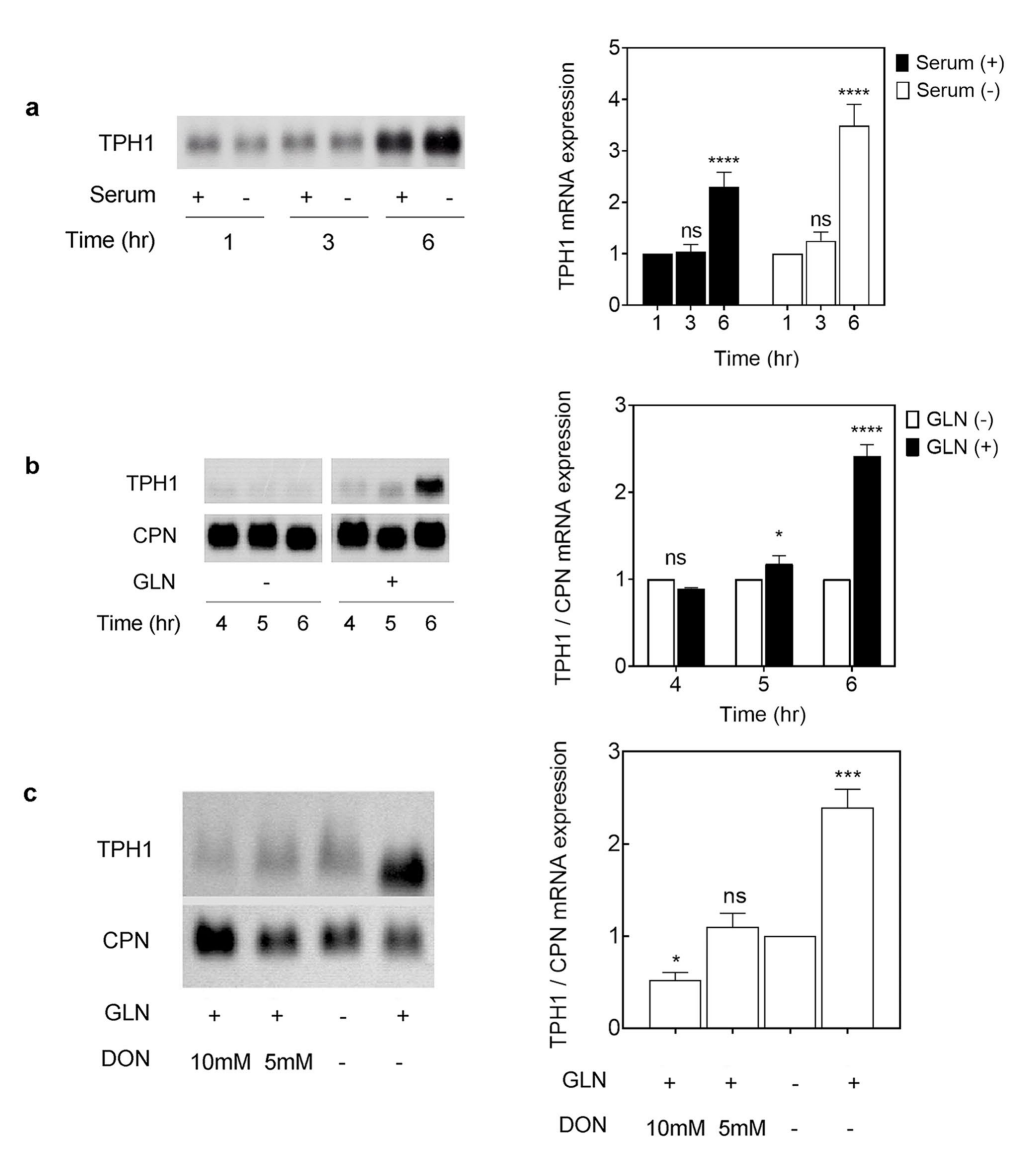
Presence of GLN elevates and anti-metabolite of GLN reduces TPH1 mRNA level irrespective of serum supplement. (A) P815-HTR cells were grown in medium containing 4 mM GLN supplemented with 10% FBS for 3-days; the cells were then rinsed and fed with serum or serum-free fresh medium. (B) After being rinsed, the cells were incubated in GLN-free medium for the indicated times and their total RNA was isolated. Equal amounts of total RNA were electrophoresed at 5 µg/lane, and Northern blotting was performed as described under in the methods section utilizing DIG-labeling probes for specific genes as indicated. Cyclophilin (CPN) was used as internal RNA standards. Histogram quantitation of the blot is shown. (C) Subconfluent P815-HTR cells were cultured in 4 mM GLN containing media and exposed to 5- or 10- mM DON (6-diazo-5-oxo-L-norleucine) for 6 h. Total RNA was extracted and separated, then examined by Northern blot analysis. The levels of CPN were used as the internal control

The P815-HTR cells were cultured in complete medium before each experiment. The subconfluent cells were re-fed with fresh culture medium containing 4 mM GLN, and the levels of TPH1 mRNA were determined at the indicated time points (Fig. 1 A and B). The medium exchange caused an increase in TPH1 mRNA levels at 6 h post treatment, mostly irrespective of presence of serum (Fig. 1 A and B). The exchange with fresh medium was thought to contain a serum-independent factor that might increase TPH1 mRNA levels. Through several experiments, it was suspected that GLN in the medium have caused the induction of TPH1 mRNA. Figure 1B shows a robust, time-dependent increase in TPH1 mRNA levels upon presence of 4 mM GLN in the medium. This effect was confirmed by treatment with the specific GLN antagonist, 6-diazo-5-oxo-L-norleucine (DON) [28], dose-dependently blocking the increases (Fig. 1 C). DON is structurally similar to GLN; due to its reactive diazo group, DON alkylates and inhibits GLN utilizing enzymes such glutaminase; it is used to block GLN dependent pathways in the cell [28]. From these results, we demonstrated that exogenous GLN induces a massive increase in TPH1 mRNA levels in P815-HTR cells (approximately 2-fold).

### Inhibition of p38 MAPK blocks the increase in TPH1 mRNA levels by GLN

**Fig. 2:**
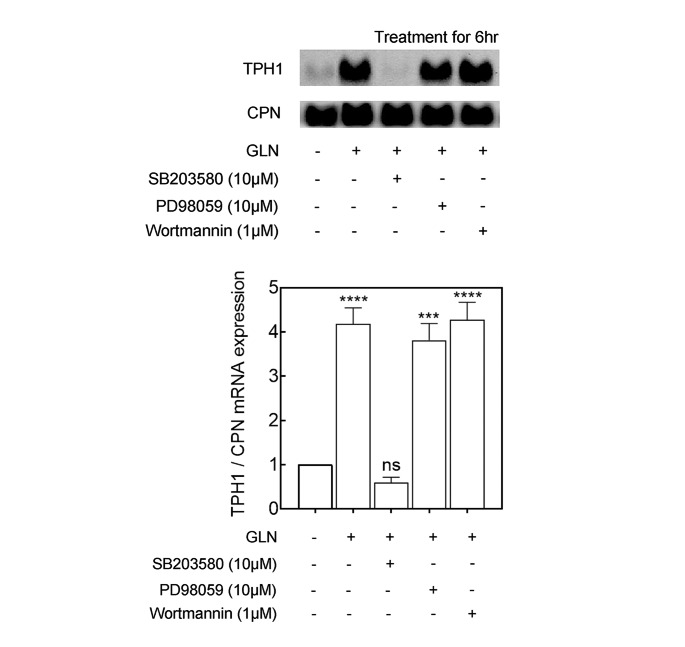
Effects of signaling pathway inhibitors on the induction of TPH1 RNA message. P815-HTR cells were grown in GLN containing medium. As indicated above, the cells were then incubated with the same fresh medium or GLN-free medium in presence of various pathway inhibitors: SB203580 (p38 MAPK inhibitor), PD98059 (p42/44 MAPK inhibitor) and wortmannin (PI3K inhibitor). After 6 h, the cells were harvested for measurement of TPH1 mRNA abundance. Total RNA was isolated from the treated cells, equal amounts of total RNA were electrophoresed (5 µg/lane), and Northern blotting was performed as described in the methods section, utilizing DIG-labeling probes for specific genes as indicated. Cyclophilin (CPN) was used as the internal RNA control

**Fig. 3:**
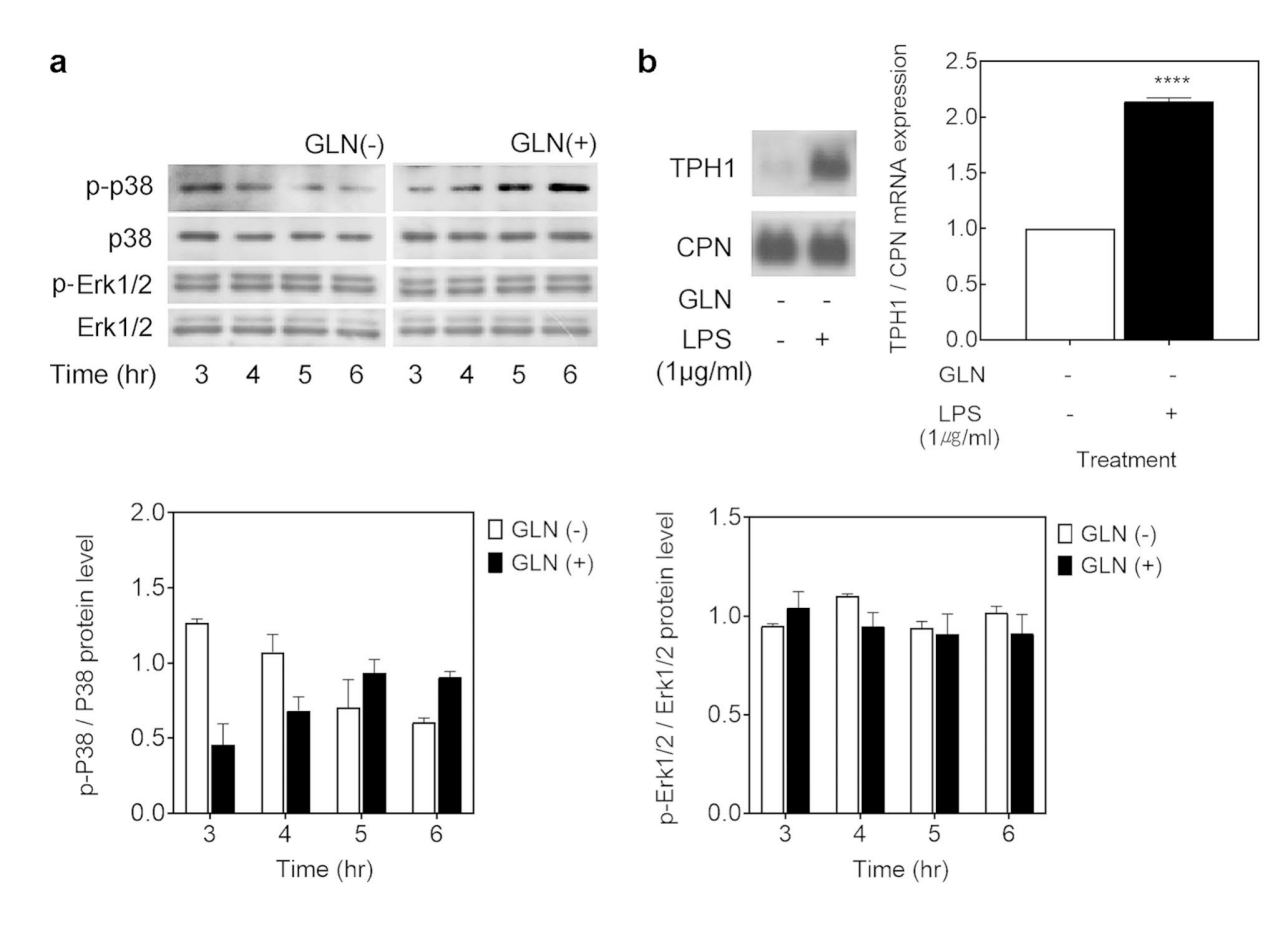
Western blot analysis for confirming that p38 kinase is involved in TPH1 mRNA induction by environmental GLN. (A) P815-HTR cells were grown in GLN containing medium. They were then fed the same fresh medium or GLN-free medium. At indicated time points, the cells were harvested for measurement of activated MAPKs. Following SDS-polyacrylamide slab gel electrophoresis, the resolved proteins were transferred onto PVDF membrane and blotted by Western probing. The probed proteins were visualized as described in the methods section. (B) After being rinsed, the cells were stimulated with bacterial lipopolysaccharide (LPS) at 1 µg/ml, a potent activator of p38 MAPK. After 6 h, the cells were harvested for measurement of TPH1 mRNA abundance. Total RNA was isolated from the cells, and equal amounts of total RNA were electrophoresed at 5 µg/lane. Northern probing was detailed in the methods section utilizing DIG-labeling probes for specific genes as indicated. Cyclophilin (CPN) was used as the internal RNA control

We next asked whether the observed increases in GLN-led TPH1 mRNA increases were dependent on MAP kinase or PI3K pathways. The agents used were SB203580 (p38 MAPK inhibitor), PD98059 (p42/44 MAPK inhibitor) and wortmannin (PI3K inhibitor) and the treated samples were compared with the control (carrier treated) TPH1 mRNA levels [29, 30]. Subconfluent cells were fed complete cultured medium, containing 4 mM GLN, in presence of SB203580, PD98059, or wortmannin for 6 h, and the changes in TPH1 mRNA levels were measured (Fig. 2). Only the p38 MAPK inhibitor, SB203580, was seen to abolish the GLN induction of TPH1 mRNA at 6 h (Fig. 2). The P815-HTR cells were then cultured for the indicated times in fresh DMEM in absence or presence of 4 mM GLN and activation of p38 kinase was confirmed by GLN treatment (Fig. 3 A; right panel). As p38 mitogen-activated protein kinase is also activated by bacterial lipopolysaccharide (LPS) [31], we examined whether P815-HTR cells activated in their p38 kinase post a 6 h exposure to LPS containing medium in absence of GLN would alter their abundance of TPH1 mRNA as measured by northern blot analysis (Fig. 3B). The above was indeed observed with the conclusion that p38 kinase activity is essential for TPH1 mRNA induction by GLN and activation of p38 MAPK pathway can substitute for the treatment of the cells by GLN.

### Transcription of TPH1 gene is increased by GLN

**Fig. 4:**
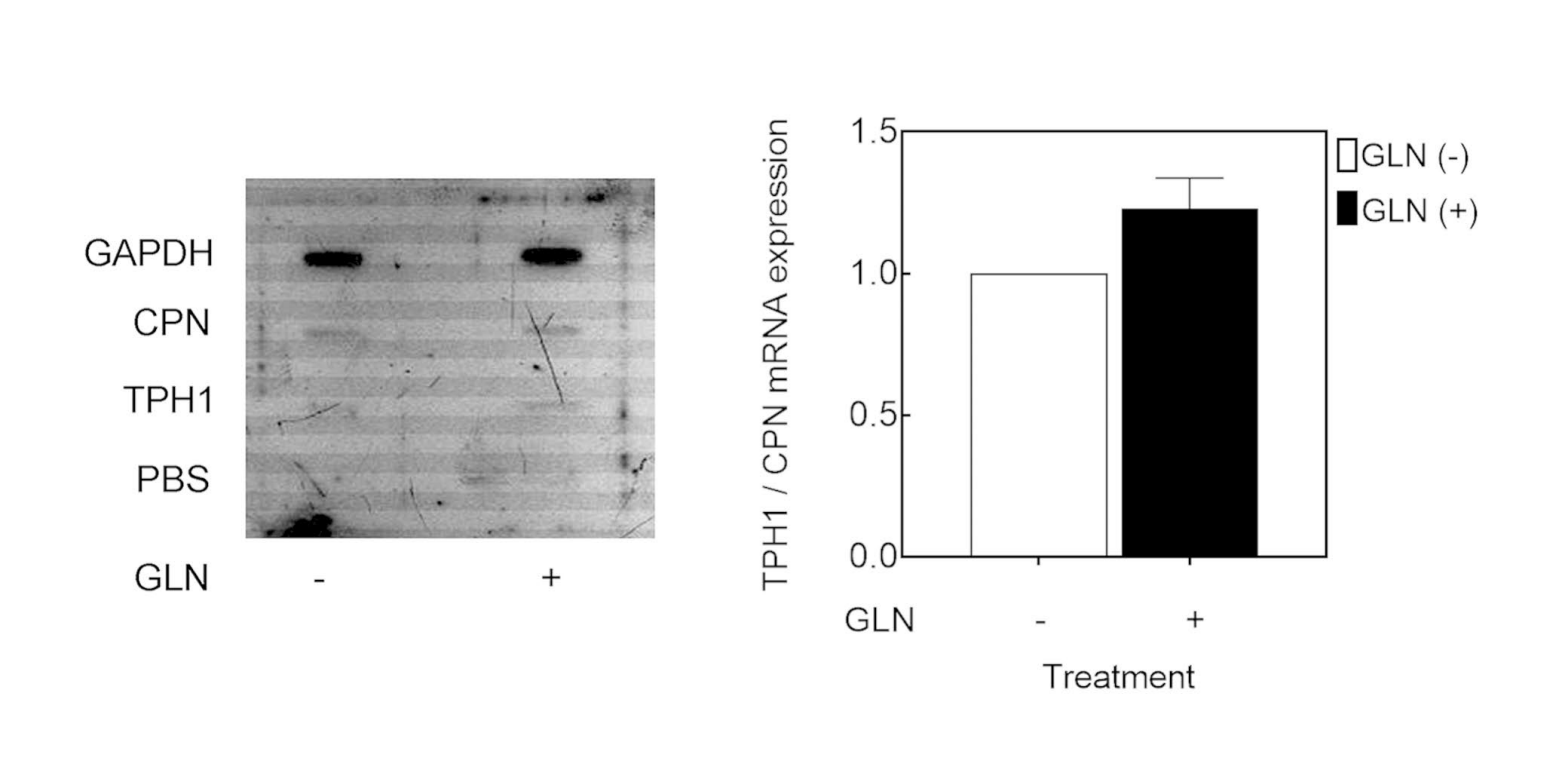
Effect of environmental GLN on TPH1 transcription rates. Subconfluent P815-HTR were cultured in medium having 4 mM GLN. The cells were then washed and incubated with medium either with or without 4 mM GLN. At 6 h post feeding, the cells were harvested, and their nuclei were isolated and cryopreserved. In a nuclear run-on assay, transcription from 30 million nuclei were allowed to proceed for 30 min at 30 °C with radio-labeled UTP. Membrane capture was via binding with 5 µg each of linearized/denatured plasmids (CPN, GAPDH, and TPH1). Two identical capture membranes were hybridized for 48 h with equal activities of 1.4 ⋅ 10^7^ cpm of RNA from cells with/without GLN; this was proceeded by washing and treatment with RNase and exposure to film for autoradiography, as described in the methods section

The increases in the levels of TPH1 mRNA due to exogenous GLN could be from a number of mechanisms, such as augmented transcription, improved RNA stability, or both. To test the effects of exogenous GLN on TPH1 gene transcription, a nuclear run-on assay was performed to determine transcription initiation rate for *TPH1* gene. Subconfluent P815-HTR cells were washed and incubated with medium in presence or absence of 4 mM GLN. Following a 5.5 h incubation, the cellular nuclei were harvested. Equivalent purified and in vitro [α-32P] UTP labeled RNA was incubated on membranes for run-on transcription of nuclei from the GLN-containing and GLN-withheld cells. The results are shown with the GAPDH housekeeping control along with cyclophilin (CPN) used as internal RNA standards (Fig. 4). For RNA derived from the nuclei of GLN-fed cells, the amount of radioactivity captured by the TPH1 cDNA was only slightly increased (by less than 0.3-fold). Similar results were obtained for three repeats. As the overall increases in TPH1 transcription by GLN were always by 2 to 3-fold, this suggested that GLN causes up-regulation of TPH1 mRNA levels by different means and not solely by increased transcription.

### TPH1 mRNA is intrinsically unstable and inhibition of p38 MAPK accelerates its decay

**Fig. 5:**
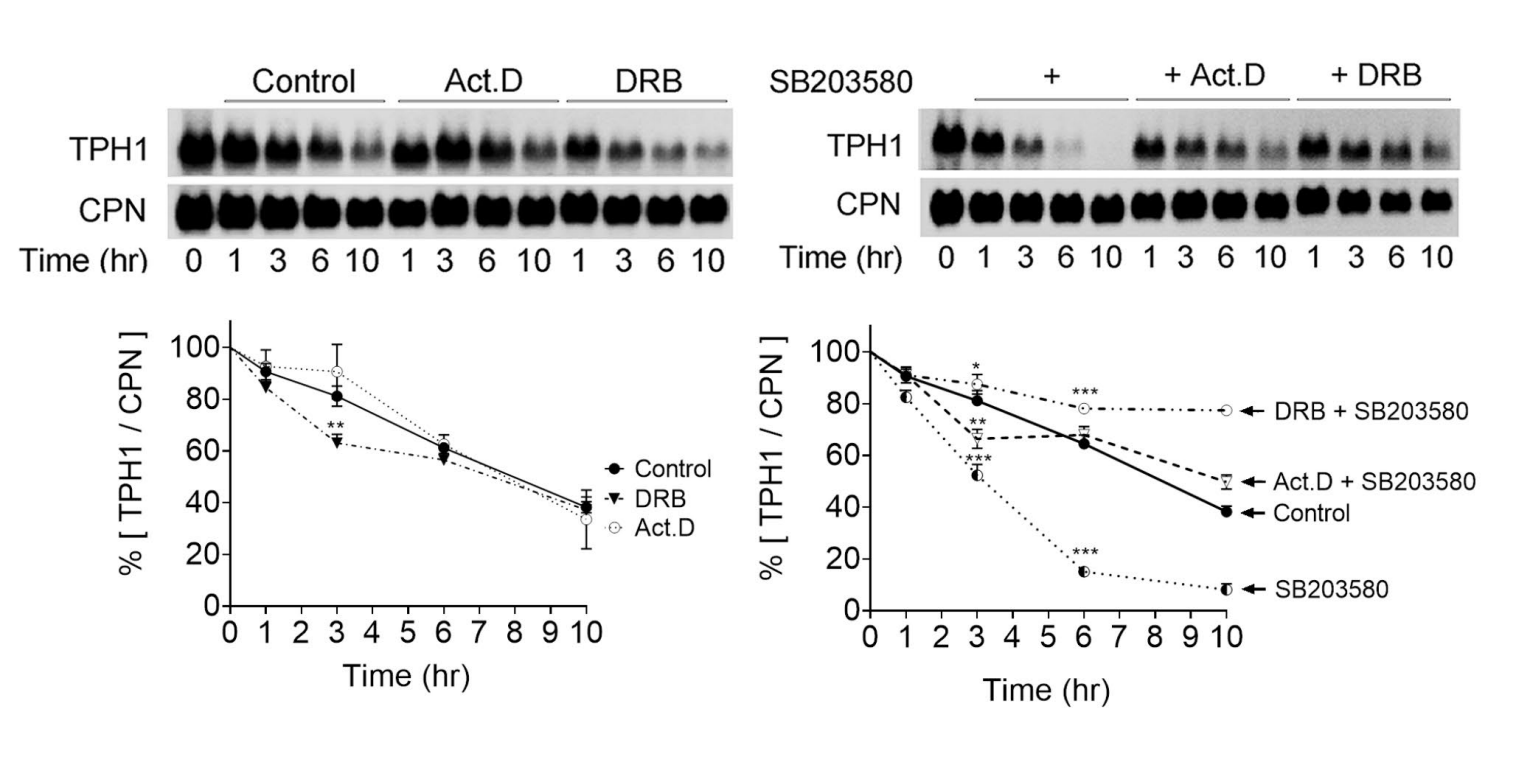
Effect of p38 MAPK inhibitor, SB203580, on TPH1 mRNA decay rates. Cells were fed with 4 mM GLN for overnight and then their culture was switched to a GLN-free one. They were also incubated with transcriptional inhibitors (actinomycin D 10 µg/ml or DRD 100 µM) or the carrier control, in a combination with 10 µM SB203580 as indicated in the figure. At the various times post treatment, the cells were harvested for measurement of TPH1 mRNA levels. Total RNA was isolated from the cells, and equal amounts of total RNA were electrophoresed (5 µg/lane), followed by Northern blotting of the samples. Relative mRNA levels were calculated and plotted by comparison with CPN-normalized at time 0, defined as the time point where the cells had been transferred to GLN-free conditions

The p38 MAPK activity in cells may modulate the half-life of TPH1 mRNA; as such, changes in the half-life of TPH1 mRNA in cells post GLN feeding were studied. Subconfluent P815-HTR cells were first fed and cultured in 4 mM GLN containing medium for approximately 14 h, followed by 10 h of monitoring their TPH-1 RNA levels after transferring them to GLN-free culture conditions (Fig. 5). The changes in the levels of mRNA were compared with that of cyclophilin (CPN) mRNA, with normalized values at various times post monitoring of mRNA. Compared with CPN RNA message, there was a steady decline in TPH1 mRNA levels in control treated cells when they were cultured in GLN-free media, and this was similar when in presence of either of two transcription inhibitors, actinomycin D or DRB (53-85-0), blocking transcription via various means [32]. This implied that the message levels for TPH1 are intrinsically unstable and they go through a fast decay when cells run down their sources of GLN (Fig. 5). Treatment of the cells with p38 MAPK inhibitor, SB203580, accelerated the decay of TPH1 mRNA, which was blunted with a global transcriptional block (SB203580 in presence of actinomycin D (Act.D) or SB203580 in presence of DRB). The following observations are made: (1) The rate of SB203580-led TPH1 mRNA decay was higher than the baseline decay, with SB203580 treatment enhancing the degradation and destabilization of TPH1 mRNA. (2) Presence of Act.D or DRB treatment blunted the effect of SB203580. (3) DRB treatment had a stronger reversing effect than Act.D. (4) From these, we hypothesize that the p38 MAPK activity inhibits transcription of certain gene(s) mediating TPH1 mRNA destabilization.

## Discussion

In this report, we describe the mechanism for accumulation of TPH1 mRNA in cultured P815-HTR mouse mastocytoma cells; it should be noted that TPH enzymatic activity in P815-HTR cells has been previously reported [33]. Due to the unavailability of a TPH-positive cell line with a pineal or neuronal lineage, the P815 and P815-HTR (high transfection) lines were used for analysis of TPH1 transcription regulation and the factors affecting its RNA half-life. We showed that exchange of culture medium stimulated the induction of TPH1 mRNA. This effect was independent of presence of serum and depended on presence of exogenous GLN in the media of the cultured cells (Fig. 1 A).

GLN is a required nutrient for cell manufacture of purines and pyrimidines and also a usable energy source for the cell, shown for cultured mammalian cells and also many tumor cells [34] as GLN plays a key role in tumor cell metabolism, with multiple tumor types using GLN as their major respiratory fuel [35]. We hypothesized that the cellular GLN might be important in regulating TPH1 mRNA expression in P815-HTR cells. Consistent with this view, use of a glutamine antagonist, 6-diazo-5-oxo-L-norleucine (DON), suppresses the TPH1 mRNA induction by exogenous GLN (Fig. 1B, C). DON is a glutamine anti-metabolite and it blocks mitochondrial glutaminases and amidotransferases that are dependent on glutamine.

We found p38 MAPK activity to be increased by GLN treatment of P815-HTR cells (Fig. 2) and was essential for stability of TPH1 mRNA in those cells (Fig. 5). Similarly, p38 MAPK has been documented to be involved in Cox-2 mRNA stability [36]. In those reports, p38-dependent Cox-2 mRNA stabilization was via the p38 substrate MAPKAPK-2 and was probably in part due to the small heat shock protein hsp27 becoming phosphorylated. The increased RNA half-life due to p38 MAPK was considered to be gene specific, as 3’ UTR sequences derived from c-myc or TNFα also destabilized the β-globin reporter transcript but were not responsive to the p38 MAPK signaling [36]. The p38 pathway has also been shown to regulate the half-life of reporter transcripts having GM-CSF, c-fos, IL-6, and IL-8 response elements (ARE) [37, 38].

In this study, we demonstrate that p38 kinase activation elicits a substantial increase in the stability of TPH1 mRNA. But the putative AREs do not exist in TPH1 3’UTR for p38-mediated stability of TPH1 message; therefore, it seems that they are distinct mechanisms other than cis-acting sequences in regulating TPH1 mRNA stability. In our study, transcription of the stability of TPH1 mRNA was studied in presence or absence of actinomycin D or DRB transcription inhibitors [32]. The chance of actinomycin D with DRB directly modulating the processes in TPH1 mRNA decay could not be excluded, as transcriptional inhibitors may affect the nuclear-to-cytoplasmic trafficking of a number of RNA binding proteins [39–41], and directly affect the stability of certain mRNA species [42]. We do not believe that this was the case in our study, as the rate of actinomycin D or DRB treated samples for TPH1 mRNA decay were similar to that of their intrinsic RNA decay (Fig. 5). Another caveat in this study was that at high concentrations, SB203580 may inhibit some JNK activity [43]; however, activation of JNK by GLN was not observed (data not shown). In addition, beyond the transcriptional and RNA stability regulation of TPH1 message, we did not assay TPH1 protein level changes or changes in TPH1 enzymatic activity with respect to exogenous glutamine levels as these will need to be described in a future study and in more target tissues beyond what we see in P815-HTR cells. The study would also be required to discover a role, if any, for physiological changes in glutamine and how they affect the total activity of TPH1 in various tissues.

On the role of environmental GLN in regulating the levels and activity of TPH1, we hypothesize that exogenous GLN via TPH1 physiologically affects the stores of serotonin and its metabolites [6]. These may have profound effects as there are a myriad of functions in the periphery for serotonin [3]. GLN levels are also modulated in feeding, starvation, health and disease [22], and these may lead to serotonin level changes. Interestingly, as the blood–brain barrier blocks the ready traverse of serotonin into and out of the CNS, the peripheral and central serotonin compartments are thought to be functionally separate and any systemic changes of GLN may have varying effects on a given compartment of serotonin [44]. Serotonin is also used in serotonylation of various proteins such as fibronectin, certain GTPase proteins, and histones [3]. This also adds another layer of complexity to the physiological effects of GLN changes on cellular substrates of serotonin. All of these areas need to be addressed in animal studies for physiological changes due to GLN that affect serotonin.

## Conclusions

The details of environmental GLN and subsequent signaling for transcriptional increase and stabilization of TPH1 mRNA remain to be addressed and are deemed to be significant as dramatically up-regulated levels of TPH1 message represent a potential mechanism for the long observed and yet poorly understood GLN effects on various mammalian cells.

## Electronic supplementary material

Below is the link to the electronic supplementary material.

Supplementary Material 1         None

Supplementary Material 2          None
