# Supplementary material for: Glutamine increases stability of TPH1 mRNA via p38 mitogen-activated kinase in mouse mastocytoma cells
Source: Mol Biol Rep. 2022 Nov 4;50(1):267–77. doi: 10.1007/s11033-022-07693-7 (PMC9884262; doi:10.1007/s11033-022-07693-7)
Supplement: Supplementary file 1 — Supplementary Material 1 None [file 11033_2022_7693_MOESM1_ESM.pdf]

CERTIFICATE OF ENGLISH EDITING

SynergyBio

SynergyBio Editing

This certificate confirms that the manuscript listed below was edited by one or more expert editors with a PhD, being native English speakers.
The following issues were corrected: grammar, spelling, punctuation, sentence structure, usage and phrasing.

Journal editors can contact us for a copy of the edited document that was submitted to the authors.

Manuscript title:

"Glutamine increases stability of TPH1 mRNA via p38 mitogen-activated kinase in mouse mastocytoma cells"

by Park et al.

Date issued:

June 3, 2022

Certificate number:

20220603A

SynergyBio Editing provides editing, proofreading, and formatting services for technical manuscripts, grant applications, conference presentations and posters, and professional school application essays and other scientific materials. All orders are edited by one or more professional scientific editors who hold advanced PhD degrees and are native English speakers.
