# Supplementary material for: Glutamine increases stability of TPH1 mRNA via p38 mitogen-activated kinase in mouse mastocytoma cells
Source: Mol Biol Rep. 2022 Nov 4;50(1):267–77. doi: 10.1007/s11033-022-07693-7 (PMC9884262; doi:10.1007/s11033-022-07693-7)
Supplement: Supplementary file 2 — Supplementary Material 2 None [file 11033_2022_7693_MOESM2_ESM.pdf]

# 한림대학교
# 카피킬러캠퍼스 표절 검사
# 결과 확인서

| 확 인 |
| --- |
| 성 명 |
| 서 명 |

| | | | |
| --- | --- | --- | --- |
| 아이디 | 42553 | 표절률 | 13% |
| 소속 | 자필로 기재하세요 | | |
| 성명 | 자필로 기재하세요 | | |

| | | | |
| --- | --- | --- | --- |
| 검사번호 | 00175334748 | 검사일자 | 2022.05.14 13:19 |
| 발급형태 | ▫ 기본보기 ▫ 요약보기 ■ 상세보기 | 발급일자 | 2022.05.14 13:19 |
| 검사명 | Heeyoung Park | | |
| 문서명 | Tracked Version.docx | | |
| 비고 | | | |

| | |
| --- | --- |
| 비교범위 | [현재첨부문서] [카피킬러 DB] |
| 검사설정 | 표절기준 [6 어절], 인용/출처 표시문장 [제외], 법령/경전 포함문장 [제외], 목차/참고문헌 [제외] |

| | |
| --- | --- |
| 검토 의견 | |

Copyright ⓒ MUHAYU All Rights reserved.

## 분석 정보

| 표절률 | 전체문장 | 동일문장 | 의심문장 | 인용/출처 | 법령/경전 |
|---|---|---|---|---|---|
| 13% | 146 | 2 | 31 | 1 | 0 |

## 비교 문서 정보

| 번호 | 표절률 | 출처정보 | 비고 |
|---|---|---|---|
| 1 | 8% | [카피킬러 DB] Copykiller<br>- 파일명 : Studies on the tryptophan hydroxylase gene expression in mouse mastocytoma cell line, P815-HTR : Mouse mastocytoma cell line(P815-HTR)을 이용한 세로토닌 합성 효소 유전자(Tryptophan hydroxylase)의 발현 및 조절에 관한 연구<br>- 저자 : 이창욱<br>- 발행 : 춘천 : 한림대학교 대학원, 2000 | |
| 2 | 2% | [카피킬러 DB] Copykiller<br>- 파일명 : Isovaleric acid ameliorates ovariectomy-induced osteoporosis by inhibiting osteoclast differentiation = 이소발레르산의 파골 세포 분화 억제를 통한 골다공증 개선 효과 (다운로드)<br>- 저자 : Kwang Min Cho Isovaleric acid ameliorates ovariectomy-induced osteoporosis by inhibiting osteoclast differentiation = 이소발레르산의 파골 세포 분화 억제를 통한 골다공증 개선 효과 | |
| 3 | 1% | [카피킬러 DB] Copykiller<br>- 파일명 : Catchin, a novel protein in molluscan catch muscles, is produced by alternative splicing from the myosin heavy chain gene<br>- 저자 : Akira Yamada; Maki Yoshio; Kazuhiro Oiwa; László Nyitray<br>- 발행 : 2000 | |
| 4 | 1% | [카피킬러 DB] Copykiller<br>- 파일명 : Roles of cAMP in regulation of tryptophan hydroxylase(TPH) gene expression in serotonergic CA77 cells = 세로토닌성 CA77세포주에서 cAMP에 의한 Tryptophan Hydroxylase(TPH) 유전자 조절에 관한 연구<br>- 저자 : 權龍俊<br>- 발행 : 춘천: 翰林大學校, 2004 | |
| 5 | 1% | [카피킬러 DB] Copykiller<br>- 파일명 : Roles of cAMP in regulation of tryptophan hydroxylase(TPH) gene expression in serotonergic CA77 cells = 세로토닌성 CA77세포주에서 cAMP에 의한 Tryptophan Hydroxylase(TPH)유전자 조절에 관한 연구<br>- 저자 : 권용준<br>- 발행 : 춘천 : 한림대학교 대학원, 2005.2 | |
| 6 | 1% | [카피킬러 DB] www.ncbi.nlm.nih.gov<br>- 파일명 : Time-restricted feeding is a preventative and therapeutic ...<br>- 발행 : www.ncbi.nlm.nih.gov | |
| 7 | 1% | [카피킬러 DB] www.nature.com<br>- 파일명 : ShadowR: a novel chromoprotein with reduced non-specific binding ...<br>- 발행 : www.nature.com | |
| 8 | 1% | [카피킬러 DB] pubmed.ncbi.nlm.nih.gov<br>- 파일명 : Time-restricted feeding is a preventative and therapeutic ...<br>- 저자 : Chaix A;Zarrinpar A;Miu P;Panda S;<br>- 발행 : 12/02/2014 | |
| 9 | 1% | [카피킬러 DB] Copykiller<br>- 파일명 : Farnesylation-defective Rheb Increases Axonal Length Independently of mTORC1 Activity in Embryonic Primary Neurons<br>- 저자 : Seunghyuk Choi, Ali Sadra, Kangjieun, Ryu, Jae Ryun, Kim, June Hoan, 선웅, 허성오<br>- 발행 : 2019, vol.28, no.2, pp. 172-182 (11 pages) | |
| 10 | 1% | [카피킬러 DB] www.koreascience.or.kr<br>- 파일명 : HeLa E-Box Binding Protein, HEB, Inhibits Promoter Activity of the Lys....<br>- 저자 : Kim, Nam-Ho;Sadra, Ali;Park, Hee-Young;Oh, Sung-Min;Chun, Jerold;Yoon, Jeong Kyo;Huh, Sung-Oh;<br>- 발행 : 2019 | |

Copyright © MUHAYU All Rights reserved.

11 1% [카피킬러 DB] www.nature.com
- 파일명 : Antifungal drug miconazole ameliorated memory deficits in a mouse ...
- 저자 : In Jun Yeo, Jaesuk Yun, Dong Ju Son, Sang-Bae Han, Jin Tae Hong
- 발행 : 2020/08/14

12 1% [카피킬러 DB] www.ncbi.nlm.nih.gov
- 파일명 : Antifungal drug miconazole ameliorated memory deficits in a mouse ... www.ncbi.nlm.nih.gov > pmc > articles > PMC7429861
- 저자 : In Jun Yeo, Jaesuk Yun, Dong Ju Son, Sang-Bae Han, Jin Tae Hong
- 발행 : August 2020

13 1% [카피킬러 DB] Copykiller
- 파일명 : Insulin-activated store-operated Ca 2+ entry via Orai1 induces podocyte actin remodeling and causes proteinuria (T202105338.pdf)
- 저자 : Ji-Hee Kim Kyu-Hee Hwang Bao T N Dang Minseob Eom In Deok Kong Yousang Gwack Seyoung Yu Heon Yung Gee Lutz Birnbaumer Kyu-Sang Park Seung-Kuy Cha
- 발행 : 2021-11

14 1% [카피킬러 DB] Copykiller
- 파일명 : [논문]관상동맥질환자에 있어 수술 전 brain natriuretic peptide 농도, 심장표지자, 수술전후기 변수들 간의 상관관계와 임상적 유용성
- 저자 : 생명과학회지 = Journal of life science v.20 no.9 = no.125 , 2010년, pp.1299 - 1305 최석철 (부산가톨릭대학교 보건과학대학 임상병리학과 ) ; 김양원 ( 인제대학교 의과대학 부산백병원 응급의학과 ) ; 현경예 ( 동의대학교 자연과학대학 임상병리학과 ) ; 황수명 ( 부산가톨릭대학교 보건과학대학 임상병리학과 ) ; 문성민 ( 인제대학교 식의학생명공학대학 임상병리학과)
- 발행 : 2010

15 1% [카피킬러 DB] Copykiller
- 파일명 : Prostate epithelial genes define therapy-relevant prostate cancer molecular subtype (T202105726.pdf)
- 저자 : Hyunho Han Hyung Ho Lee Kwibok Choi Young Jun Moon Ji Eun Heo Won Sik Ham Won Sik Jang Koon Ho Rha Nam Hoon Cho Filippo G Giancotti Young-Deuk Choi
- 발행 : 2021-12

16 1% [카피킬러 DB] Copykiller
- 파일명 : Studies on the mechanisms of signal transduction pathway by bioactive lysophospholipids in cultured mammalian cells = 동물배양세포에서 생활성 라이소지질에 의한 신호전달 기전에 관한 연구
- 저자 : 이창욱
- 발행 : 춘천 : 한림대학교 대학원, 2004.2

17 1% [카피킬러 DB] Copykiller
- 파일명 : Studies on the mechanisms of signal transduction pathway by bioactive lysophospholipids in cultured mammalian cells = 동물배양세포에서 생활성 라이소지질에 의한 신호전달 기전에 관한 연구
- 저자 : 李昌旭
- 발행 : 춘천: 한림대학교, 2003

18 1% [카피킬러 DB] Copykiller
- 파일명 : Role of posttranslational modification of Ras-homologue-enriched-in-brain (Rheb) in determination of axonal length of mammalian embryonic neurons = 포유동물 배아뇌신경세포의 축삭 길이 결정에 관여하는 Rheb 단백질의 분자·세포생물학적 기전 연구
- 저자 : 최승혁
- 발행 : Thesis(Ph.D.) -- Graduate School, Hallym University Major in Pharmacology, Department of Medical Science 2019

19 1% [카피킬러 DB] Copykiller
- 파일명 : Role of posttranslational modification of Ras-homologue-enriched-in-brain (Rheb) in determination of axonal length of mammalian embryonic neurons = 포유동물 배아뇌신경세포의 축삭 길이 결정에 관여하는 Rheb 단백질의 분자·세포생물학적 기전 연구
- 저자 : 최승혁
- 발행 : 2019

20 1% [카피킬러 DB] Copykiller
- 파일명 : Roles of cAMP in regulation of tryptophan hydroxylase(TPH) gene expression in serotonergic CA77 cells = 세로토닌성 CA77세포주에서 cAMP에 의한 Tryptophan Hydroxylase(TPH)유전자 조절에 관한 연구
- 저자 : 권용준
- 발행 : 춘천 : 한림대학교 대학원, 2005.2

21 1% [카피킬러 DB] Copykiller

22 1% [카피킬러 DB] Copykiller
- 파일명 : Defective neuronal migration and inhibition of bipolar to multipolar transition of migrating neural cells by Mesoderm-Specific Transcript, Mest, in the developing mouse neocortex
- 저자 : Ji, Liting; Bishayee, Kausik; Sadra, Ali; Choi, Seunghyuk; Choi, Wooyul; Moon, Sungho; Jho, Eek-hoon; Huh, Sung-oh
- 발행 : 2017-5

Copyright © MUHAYU All Rights reserved.

| | | |
|---|---|---|
| 23 | 1% | [카피킬러 DB] link.springer.com<br>- 파일명 : N , N -disubstituted azines attenuate LPS-mediated ...<br>- 저자 : Lalita Subedi, Oh Wook Kwon, Chaeho Pak, Goeun Lee, Kangwoo Lee, Hakwon Kim, Sun Yeou Kim<br>- 발행 : 2017/12/28 |
| 24 | 1% | [카피킬러 DB] synapse.koreamed.org<br>- 파일명 : Anti-Inflammatory Effect of Violapyrones B and C from a Marine ... synapse.koreamed.org > articles<br>- 저자 : Hwa-Sun Lee, Bong-Jeun An, Hyeon Jeong Kim, Yong Hun Cho, Dong In Kim, Jae Yoon Jang, Jae Hoon Kwak, Hyi-Seung Lee, Yeon-Ju Lee, Jong Seok Lee, Hee Jae Shin<br>- 발행 : 2015/12/31 |
| 25 | 1% | [카피킬러 DB] www.ncbi.nlm.nih.gov<br>- 파일명 : Synergistic anti-inflammatory effects of Nobiletin and Sulforaphane ... www.ncbi.nlm.nih.gov > pmc > articles > PMC3296826<br>- 저자 : Shanshan Guo, Peiju Qiu, Guang Xu, Xian Wu, Ping Dong, Guanpin Yang, Jinkai Zheng, David Julian McClements, Hang Xiao<br>- 발행 : 7 March 2012 |
| 26 | 1% | [카피킬러 DB] Copykiller<br>- 파일명 : Anti-osteoporotic effects ofPueraria candolleivar.mirificaon bone mineral density and histomorphometry in estrogen-deficient rats<br>- 저자 : Suthon, Sarocha; Jaroenporn, Sukanya; Charoenphandhu, Narattaphol; Suntornsaratoon, Panan; Malaivijitnond, Suchinda<br>- 발행 : 2016-4 |
| 27 | 1% | [카피킬러 DB] Copykiller<br>- 파일명 : Changes in self-rated health and quality of life among Syrian refugees migrating to Norway: a prospective longitudinal study<br>- 저자 : [Author(affiliation=Department of Global Public Health and Primary Care, University of Bergen, name=Jasmin Haj-Younes), Author(affiliation=Department of Global Public Health and Primary Care, University of Bergen, name=Elisabeth Marie Strømme), Author(affiliation=Department of Global Public Health and Primary Care, University of Bergen, name=Jannicke Igland), Author(affiliation=Unit for Migration and health. Norwegian Institute of Public Health, name=Bernadette Kumar), Author(affiliation=Department of Psychosocial Health, University of Agder, name=Eirik Abildsnes), Author(affiliation=Department of Global Public Health and Primary Care, University of Bergen, name=Wegdan Hasha), Author(affiliation=Department of Global Public Health and Primary Care, University of Bergen, name=Esperanza Diaz)]<br>- 발행 : 2020-11-01T12:26:16Z |
| 28 | 1% | [카피킬러 DB] Copykiller<br>- 파일명 : Tobacco smoking and the risk of gallbladder disease<br>- 저자 : Dagfinn Aune / Lars J. Vatten / Paolo Boffetta<br>- 발행 : 2016/02/22 |
| 29 | 1% | [카피킬러 DB] Copykiller<br>- 파일명 : Polymyositis with mitochondrial pathology or atypical form of sporadic inclusion body myositis: case series and review of the literature<br>- 저자 : George K. Papadimas<br>- 발행 : 2019 5 4 |
| 30 | 1% | [카피킬러 DB] behavioralandbrainfunctions.biomedcentral.com<br>- 파일명 : The neural correlates of mental arithmetic in adolescents: a ...<br>- 발행 : behavioralandbrainfunctions.biomedcentral.com |

Copyright © MUHAYU All Rights reserved.

| 검사 문서 | 비교 문서 |
| --- | --- |

문장표절률: 0%

Glutamine increases stability of TPH1 mRNA via p38 mitogen-activated kinase in mouse mastocytoma cells Heeyoung Park†, Chang-Wook Lee†, Jieun Kang, Ali Sadra and Sung-Oh Huh* († These authors contributed equally. * Corresponding author)

문장표절률: 67%

Author identifying information Affiliation: Department of Pharmacology, College of Medicine, Institute of Natural Medicine, Hallym University, Chuncheon 24252, Gangwon-Do South Korea Corresponding author: ProfessorSung-Oh Huh. Phone: +82-33-248-2615; Fax: +82-33-248-3188 E-mail: s0huh@hallym.ac.kr

[www.koreascience.or.kr] HeLa E-Box Binding Protein, HEB, Inhibits Promoter Activity of the Lys....

저자 : Kim, Nam-Ho;Sadra, Ali;Park, Hee-Young;Oh, Sung-Min;Chun, Jerold;Yoon, Jeong Kyo;Huh, Sung-Oh;
발행 : 2019

Neuroblast Cells Kim, Nam-Ho Department of Pharmacology, College of Medicine, Institute of Natural Medicine, Hallym University) ; Sadra, Ali Department of Pharmacology, College of Medicine, Institute of Natural Medicine, Hallym University) ; Park, Hee-Young Department of Pharmacology, College of Medicine, Institute of Natural Medicine, Hallym University) ; Oh, Sung-Min Department of Pharmacology, College of Medicine, Institute of Natural Medicine, Hallym University Chun, Jerold (Sanford Burnham Prebys ...... Soonchunhyang University) ; Huh, Sung-Oh Department of Pharmacology, College of Medicine, Institute of Natural Medicine, Hallym University) Received : 2018.10.02 Accepted

[www.ncbi.nlm.nih.gov] Angelica polymorpha Maxim Induces Apoptosis of Human SH-SY5Y ... www.ncbi.nlm.nih.gov > pmc > articles > PMC4757799

저자 : Md. Ataur Rahman, Kausik Bishayee, Sung-Oh Huh
발행 : 29 February 2016

Copyright and License information Disclaimer Department of Pharmacology, College of Medicine, Institute of Natural Medicine, Hallym University, Chuncheon 200-702, Korea Correspondence: rk

문장표절률: 0%

ORCID ID: Heeyoung Park (H.P.): 0000-0002-6939-7160, Chang-Wook Lee (C.W.L.): 0000-0002-0907-8475; Jieun Kang (J.K.): 0000-0003-0556-4422, Ali Sadra (A.S.): 0000-0002-1938-1072; Sung-Oh Huh (S-O.

문장표절률: 0%

H.): 0000-0002-6019-6450 Conflict of Interest: Sung-Oh Huh has received research grants from Hallym University Research Fund, declares that he has no conflict of interest. All the authors declare that they have no conflict of interest.

문장표절률: 0%

Running Title Glutamine-mediated regulation of TPH1 mRNA stability Glutamine increases stability of TPH1 mRNA via p38 mitogen-activated kinase in mouse mastocytoma cells Abstract Expression changes for tryptophan hydroxylase 1 (TPH1), the rate-limiting enzyme in serotonin synthesis, by environmental glutamine (GLN) were examined in mouse mastocytoma-derived P815-HTR cells.

문장표절률: 0%

GLN-treated cells exhibited a robust increase in TPH1 mRNA after a 6 hr exposure to GLN. 6-Diazo-5-oxo-L-norleucine (DON), a glutamine-utilizing glutaminase inhibitor, significantly inhibited the GLN-induction of TPH1 mRNA.

문장표절률: 0%

Nuclear run-on assays and mRNA decay experiments demonstrated that the primary mechanism leading to increased TPH1 mRNA levels was not due to transcriptional changes, but rather due to increased TPH1 RNA stability induced by GLN.

문장표절률: 0%

Treatment with GLN also led to activation of p38 MAP kinase, but not p42/44 MAPK. In addition, SB203580, a p38 MAP kinase specific inhibitor, completely abolished the GLN-mediated increase of TPH1 mRNA levels, suggesting the pathway stabilizing TPH1 mRNA might be mediated by the activated p38 MAP kinase pathway.

Copyright © MUHAYU All Rights reserved.

문장표절률: 0%

Additionally, SB203580 significantly reduced the stability of TPH1 mRNA, and this reduction of thestability was not affected by GLN in the culture medium, im plying a sequential signaling from GLN being mediated by p38 MAP kinase, res ulting in alteration of TPH1 mRNA stability.

문장표절률: 0%

TPH1 mRNA stability loss was also dependent on de novo protein synthesis as shown by treatment of cells with a transcriptional/translational blocker.

문장표절률: 0%

We provide evidence that TPH1 mRNA levels are increased in response to incre ased exogenous GLN in mouse mastocytoma cells via a stabilization of TPH1 mRNA due to the activity of the p38 MAP kinase.

문장표절률: 0%

Keywords: Tryptophan hydroxylase 1; Glutamine; p38 MAP kinase; mRNA sta bility Introduction As a neurotransmitter in the CNS, serotonin or 5-hydroxytry ptamine (5-HT), has roles in a number of behavioral disorders such as depressi on, substance abuse, schizophrenia and autism [1].

문장표절률: 0%

In the CNS, the primary source of serotonin are the serotonergic neurons. How ever, the majority of serotonin is outside of the CNS, with the enterochromaffin (EC) cells of gastrointestinal epithelial tract making about 90% of serotonin in th e body [2].

문장표절률: 0%

The roles of serotonin in the periphery are quite diverse, such as being involved i n gastrointestinal functions, production of insulin, cell bioenergetics, bone home ostasis, and blood coagulation.

문장표절률: 0%

Serotonin has also been detected as being covalently integrated into proteins in a process called serotonylation [3]. Many substrates of serotonylation have been i dentified; these include extracellular matrix components such as fibronectin and certain GTPase proteins.

문장표절률: 0%

The discovered serotonylation dependent activities are numerous including contr action of smooth muscle and activation of platelets [4, 5].

문장표절률: 0%

Serotonin is the precursor to N-acetylserotonin (NAS) and melatonin, both with their own biological functions. Serotonin and NAS are also detectable in serum, and these serum supplies are conjectured to serve as substrates for production o f melatonin [6, 7].

문장표절률: 39%

The rate-limiting step in serotonin synthesis is catalyzed by tryptophan hydroxyl ase (TPH), with TPH activity detected in serotonergic neurons in the CNS, pine al gland, retina, gut intestinal and pancreatic EC cells [8].

[scienceon.kisti.re.kr] 세로토닌 신경전달물질 합성의 새로운 조절기전에 관한 연구

저자 : 허성오
발행 : 2004

compulsive disorder, schizophrenia, and alcoholism. The rate-limiting step in ser otonin synthesis is catalyzed by tryptophan hydroxylase (TPH) enzyme. P815. a transformed mouse

[Copykiller] Studies on the tryptophan hydroxylase gene expression in mouse mastocytoma cell line, P815-HTR : Mouse mastocytoma cell line(P815-HT R)을 이용한 세로토닌 합성 효소 유전자(Tryptophan hydroxylase)의 발현 및 조절에 관한 연구

저자 : 이창욱
발행 : 춘천 : 한림대학교 대학원, 2000

The rate-limiting step in serotonin synthesis is catalyzed by tryptophan hydroxyl ase (TPH) enzyme (Grahame-Smith 1964). The

Copyright © MUHAYU All Rights reserved.

문장표절률: 0%

TPH activity is also detected in skin keratinocytes, fibroblasts and melanocytes [9]. In mammals, there are two distinct genes with tryptophan hydroxylase activi ty, TPH1 and TPH2.

문장표절률: 0%

TPH1 is expressed in most of the cell types mentioned above for TPH activity; on the other hand, TPH2 is almost entirely present in the brain and the neuronal cells with two alternativesplice variants described for it [10-12].

문장표절률: 0%

Little is known about the regulation of TPH1 gene expression, although a few tr anscriptional mechanisms for TPH1 have been described [13-16].

문장표절률: 0%

Difficulties in the study of TPH1 have concerned the relatively low levels of TP H1 in various tissues and its inherent instability.

문장표절률: 0%

For P815-HTR cells, a transformed mouse mastocytoma cell line, there are rea sonable levels of TPH1, allowing a study of TPH1 message regulation and the cells being used as a source of TPH1 [17].

문장표절률: 0%

TPH1 is the predominant form of the enzyme expressed in P815-HTR cells, hav ing a molecular weight of 51 kDa [18].

문장표절률: 0%

Environmental glutamine (GLN) is a major metabolite, known to affect a myriad of pathways in the cell as the result of its role in cell metabolism [19], and a role for exogenous GLN in regulation of serotonin synthesis via regulation of TPH1 levels was sought.

문장표절률: 0%

Although GLN can be manufactured by the cell, the majority of cells require exo genous GLN as it is also the most abundant amino acid in the bloodstream [19].

문장표절률: 0%

In cultured cells, for example, millimolar levels of GLN in themedia are required for survival. In the body, circulating GLN is supplied mostly by the liver, lung, a dipocytes and skeletal muscle.

문장표절률: 0%

These cells produce GLN by various means of cellular synthesis and break dow n of protein, and their contributions to the levels of circulating GLN are modifie d in various physiological states such as feeding and starvation [20].

문장표절률: 0%

Thus, environmental GLN may play a key physiological role in regulating seroto nin levels via TPH1.

문장표절률: 0%

We demonstrate that TPH1 mRNA is augmented upon exposure to exogenous GLN in mouse mastocytoma cells; this was via stabilization of TPH1 mRNA.

문장표절률: 0%

Regulation of mRNA turnover such as with RNA stability is important in regulati on of gene expression in various organisms for issues where the levels of a give n mRNA can dramatically changedue to its instability [21] and various cellular p rocesses are regulated by changes in mRNA half-life [22].

Copyright © MUHAYU All Rights reserved.

문장표절률: **0%**

We also investigated the effects of p38 MAPK activity on TPH1 mRNA levels in the P815-HTR cells in presence of exogenous GLN, as GLN metabolism has been shown to stimulate intestinal cell MAPKs [23].

문장표절률: **0%**

For p38 MAPK, it has been shown to be induced by GLN/ glycine isosmotic cell swelling [24]. We show that for TPH1, the p38 MAPK stabilizes TPH1 mRNA and presents a major pathway in regulation of TPH1 levels.

문장표절률: **0%**

Materials and methods Cell culture P815-HTR mouse mastocytoma line was obtained from ATCC. Cell culture media and chemicals werepurchased from Sigma-Aldrich, unless otherwise specified.

문장표절률: **67%**

The P815-HTR cells was grown in 100-mm culture dishes at 37 ℃ under a humidified atmosphere of 5% CO2 and 95% air. The cells weremaintained under culture conditions in Dulbecco's modified Eagle's medium (DMEM) supplemented with 10% fetal bovine serum, 20 µg/ml gentamicin and with or without added 4 mM L-glutamine (GLN).

[link.springer.com] N , N -disubstituted azines attenuate LPS-mediated ...

저자 : Lalita Subedi, Oh Wook Kwon, Chaeho Pak, Goeun Lee, Kangwoo Lee, Hakwon Kim, Sun Yeou Kim
발행 : 2017/12/28

incubated at 37 ℃ in a humidified atmosphere of 5% CO2 and 95% air. The cells weremaintained in Dulbecco's modified Eagle's medium (DMEM) supplemented with 10% fetal bovine serum, 100 U/mL penicillin and

[synapse.koreamed.org] Anti-Inflammatory Effect of Violapyrones B and C from a Marine ... synapse.koreamed.org > articles

저자 : Hwa-Sun Lee, Bong-Jeun An, Hyeon Jeong Kim, Yong Hun Cho, Dong In Kim, Jae Yoon Jang, Jae Hoon Kwak, Hyi-Seung Lee, Yeon-Ju Lee, Jong Seok Lee, Hee Jae Shin
발행 : 2015/12/31

Seoul, Korea). The cells weremaintained in Dulbecco's modified Eagle's medium (DMEM) supplemented with 10% fetal bovine serum (FBS) and antibiotics (100 U ...... and incubated for 4 hours at 37 ℃ under a humidified atmosphere of 5% CO2.

문장표절률: **17%**

Northern blotting For changes in the levels of TPH1 RNA message, the cells were incubated with either 6-diazo-5-oxo-L-norleucine (DON) (glutamine antagonist), SB203580 (p38 MAPK inhibitor), PD98059 (p42/44 MAPK inhibitor) or wortmannin (PI3K inhibitor) at the indicated concentrations in the figure legends for 6 hr with control media.

[Copykiller] Studies on the tryptophan hydroxylase gene expression in mouse mastocytoma cell line, P815-HTR : Mouse mastocytoma cell line(P815-HTR)을 이용한 세로토닌 합성 효소 유전자(Tryptophan hydroxylase)의 발현 및 조절에 관한 연구

저자 : 이창욱
발행 : 춘천 : 한림대학교 대학원, 2000

p38 MAPK inhibitor SB203580 As SB203580 (p38 MAPK inhibitor), PD98059 (p42/44 MAPK inhibitor) and wortmannin (PI3K inhibitor) have

문장표절률: **84%**

Total RNA was extracted and the RNA (5 g) was separated by 1% agarose/formaldehyde gel electrophoresis and transferred to a Hybond-N+ nylon membrane (Amersham Life Sciences, UK) essentially as described by Sambrook et al. (1989). Digoxigenine (DIG)-labeled RNA probes for Northern

[Copykiller] Studies on the tryptophan hydroxylase gene expression in mouse mastocytoma cell line, P815-HTR : Mouse mastocytoma cell line(P815-HTR)을 이용한 세로토닌 합성 효소 유전자(Tryptophan hydroxylase)의 발현 및 조절에 관한 연구

저자 : 이창욱
발행 : 춘천 : 한림대학교 대학원, 2000

and the RNA (5 ug) was separated by 1% agarose/formaldehyde gel electrophoresis and transferred to a Hybond-N+ nylon membrane (Amersham Life Science Ltd., UK) essentially as described by Sambrook et al. (1989). Digoxigenine (DIG)-labeled RNA probes for Northern blot analysis were synthesized with

[Copykiller] Roles of cAMP in regulation of tryptophan hydroxylase(TPH) gene expression in serotonergic CA77 cells = 세로토닌성 CA77세포주에서 cAMP에 의한 Tryptophan Hydroxylase(TPH) 유전자 조절에 관한 연구

저자 : 權龍俊
발행 : 춘천: 翰林大學校, 2004

in DEPC-sterile water. Total RNA (5 g) was separated by 1 agarose/formaldehyde gel electrophoresis and transferred to a Hybond-N+ nylon membrane (Amersham, Buckinghamshire, UK).

문장표절률: **98%**

blot analysis were synthesized with a DIG RNA Labeling Kit (Boehringer Mannheim). Hybridization of the labeled probes and conjugation with an anti-DIG antibody-alkaline phosphatase complex were carried out with a DIG Nucleic Acid Detection Kit (Boehringer Mannheim) according to the manufacturer.

[Copykiller] Studies on the tryptophan hydroxylase gene expression in mouse mastocytoma cell line, P815-HTR : Mouse mastocytoma cell line(P815-HTR)을 이용한 세로토닌 합성 효소 유전자(Tryptophan hydroxylase)의 발현 및 조절에 관한 연구

저자 : 이창욱
발행 : 춘천 : 한림대학교 대학원, 2000

blot analysis were synthesized with a DIG RNA Labeling Kit (Boehringer Mannheim). Hybridization of the labeled probes and conjugation with an anti-DIG antibody-alkaline phosphatase complex were carried out with a DIG Nucleic Acid Detection Kit (Boehringer Mannheim) according to the

[Copykiller] Catchin, a novel protein in molluscan catch muscles, is produced by alternative splicing from the myosin heavy chain gene

저자 : Akira Yamada; Maki Yoshio; Kazuhiro Oiwa; László Nyitray
발행 : 2000

for Northern blot analysis were synthesized with a DIG RNA Labeling Kit (SP6/T7) (Boehringer Mannheim). Total ...... by Sambrook et al. (1989). Hybridization of the labeled probes and conjugation with an anti-DIG antibody-peroxidase complex were carried out with a DIG Nucleic Acid Detection Kit (Boehringer Mannheim) according to the instruction manual. The peroxidase complex

문장표절률: **45%**

The phosphatase complex on the nylon membrane was visualized with the chemiluminescent substrate CSPD (Boehringer Mannheim) according to the manufacturer's recommendations.

[Copykiller] Roles of cAMP in regulation of tryptophan hydroxylase(TPH) gene expression in serotonergic CA77 cells = 세로토닌성 CA77세포주에서 cAMP에 의한 Tryptophan Hydroxylase(TPH)유전자 조절에 관한 연구

저자 : 권용준
발행 : 춘천 : 한림대학교 대학원, 2005.2

oven. After washed the membrane, the phosphatase complex on the nylon membrane was visualized with chemiluminescent substrate CSPD (Roche)

[Copykiller] Roles of cAMP in regulation of tryptophan hydroxylase(TPH) gene expression in serotonergic CA77 cells = 세로토닌성 CA77세포주에서 cAMP에 의한 Tryptophan Hydroxylase(TPH) 유전자 조절에 관한 연구

저자 : 權龍俊
발행 : 춘천: 翰林大學校, 2004

oven. After washed the membrane, the phosphatase complex on the nylon membrane was visualized with chemiluminescent substrate CSPD (Roche)

문장표절률: **73%**

Western blotting P815-HTR cells were lysed in radio-immunoprecipitation assay buffer (RIPA) buffer (50 mM Tris-HCl, pH 7.5, 150 mM NaCl, 1 mM EGTA, 1 mM EDTA, 1% Triton X-100, 1 mM Na3VO4, 5 mM NaF, and a protease inhibitor cocktail).

[www.koreascience.or.kr] HeLa E-Box Binding Protein, HEB, Inhibits Promoter Activity of the Lys....

저자 : Kim, Nam-Ho;Sadra, Ali;Park, Hee-Young;Oh, Sung-Min;Chun, Jerold;Yoon, Jeong Kyo;Huh, Sung-Oh;
발행 : 2019

1996) were lysed in lysis buffer (50 mM Tris-HCl, pH 7.5, 150 mM NaCl, 1 mM EGTA, 1 mM EDTA, 1% Triton X-100, 1 mM Na3VO4, 5 mM NaF, and protease inhibitor cocktail). After incubation

[www.linknovate.com] Profile for Thermo Fisher Scientific - Linknovate

were lysed in NP40 lysis buffer (50 mM Tris-HCl, pH 7.5, 150 mM NaCl, 1 mM EDTA, 1% NP40, 1.5 ...... protease inhibitor cocktail (Roche)) or RIPA buffer (50 mM Tris-HCl, pH 7.5, 150 mM NaCl, 1 mM EDTA, 1% NP40, 0.5

문장표절률: **0%**

A total of 30 μg of protein was separated per lane on 10% SDS-polyacrylamide gels, followed by transfer onto PVDF membranes (Millipore).

Copyright © MUHAYU All Rights reserved.

문장표절률: 38 %

The membranes were blocked in TBST (Tris-buffered saline containing 0.1% Tween-20) supplemented with 5% nonfat milk. Probing with the primary antibody was overnight at 4 ℃; the blot was washed three times with TBST, and then incubated with appropriate secondary antibody (anti-rabbit HRP).

[Copykiller] Spring viraemia of carp virus modulates p53 expression using two distinct mechanisms.

저자 : [Author(affiliation=null, name=Shun Li), Author(affiliation=null, name=Long-Feng Lu), Author(affiliation=null, name=Shu-Bo Liu), Author(affiliation=null, name=Can Zhang), Author(affiliation=null, name=Zhuo-Cong Li), Author(affiliation=null, name=Xiao-Yu Zhou), Author(affiliation=null, name=Yong-An Zhang)]
발행 : 2019-10-05T13:46:49Z

overnight at 4 ° C, washed three times with TBST, and then incubated with secondary Abs for 1 h

[Copykiller] Grass Carp Reovirus (GCRV) Giving Its All to Suppress IFN Production by Countering MAVS Signaling Transduction

저자 : [Author(affiliation=Institute of Hydrobiology, Chinese Academy of Sciences, Wuhan, China, name=Long-Feng Lu), Author(affiliation=College of Advanced Agricultural Sciences, University of Chinese Academy of Sciences, Beijing, China, name=Long-Feng Lu), Author(affiliation=Institute of Hydrobiology, Chinese Academy of Sciences, Wuhan, China, name=Zhuo-Cong Li), Author(affiliation=College of Advanced Agricultural Sciences, University of Chinese Academy of Sciences, Beijing, China, name=Zhuo-Cong Li), Author(affiliation=Institute of Hydrobiology, Chinese Academy of Sciences, Wuhan, China, name=Can Zhang), Author(affiliation=College of Advanced Agricultural Sciences, University of Chinese Academy of Sciences, Beijing, China, name=Can Zhang), Author(affiliation=Institute of Hydrobiology, Chinese Academy of Sciences, Wuhan, China, name=Xiao-Yu Zhou), Author(affiliation=College of Fisheries and Life Science, Dalian Ocean University, Dalian, China, name=Xiao-Yu Zhou), Author(affiliation=Institute of Hydrobiology, Chinese Academy of Sciences, Wuhan, China, name=Yu Zhou), Author(affiliation=College of Advanced Agricultural Sciences, University of Chinese Academy of Sciences, Beijing, China, name=Yu Zhou), Author(affiliation=Institute of Hydrobiology, Chinese Academy of Sciences, Wuhan, China, name=Jing-Yu Jiang), Author(affiliation=College of Advanced Agricultural Sciences, University of Chinese Academy of Sciences, Beijing, China, name=Jing-Yu Jiang), Author(affiliation=Institute of Hydrobiology, Chinese Academy of Sciences, Wuhan, China, name=Dan-Dan Chen), Author(affiliation=College of Advanced Agricultural Sciences, University of Chinese Academy of Sciences, Beijing, China, name=Dan-Dan Chen), Author(affiliation=Institute of Hydrobiology, Chinese Academy of Sciences, Wuhan, China, name=Shun Li), Author(affiliation=College of Advanced Agricultural Sciences, University of Chinese Academy of Sciences, Beijing, China, name=Shun Li), Author(affiliation=Institute of Hydrobiology, Chinese Academy of Sciences, Wuhan, China, name=Yong-An Zhang), Author(affiliation=College of Fisheries, Huazhong Agricultural University, Wuhan, China, name=Yong-An Zhang)]
발행 : 2020-10-26T05:16:06Z

dilution overnight at 4 ℃, washed three times with TBST, and then incubated with secondary Abs for 1 h

문장표절률: 0 %

After washing the blots three times in TBST, the protein bands were detected using a Western HRP substrate ECL kit (Luminata Forte, Millipore) and chemiluminescence imaging (Fusion FX, Vilber Lourmat).

문장표절률: 0 %

Primary antibodies were rabbit anti-phospho-p38 MAPK (Thr180/Tyr182) (Cell Signaling #9211; 1: 1000), rabbit anti-p38 MAPK (Cell Signaling #9212; 1: 1000), rabbit anti-phospho-Erk1/2 (Thr202/Tyr204) (Cell signaling #9101; 1: 1000), and rabbit anti-Erk1/2 (Cell Signaling #9102; 1: 1000). Secondary antibody was goat anti-rabbit IgG HRP (Thermo Fisher; 1: 5000).

문장표절률: 0 %

Nuclear run-on assay Nuclear run-on assay was performed to determine transcription initiation rate. The assay was performed as described by Greenberg and Bender (Greenberg, M.

문장표절률: 86 %

E., and Bender, T. P., 1996, in Current Protocols in Molecular Biology) and using a modification of published methods (Abcouwert, S.

[Copykiller] Studies on the tryptophan hydroxylase gene expression in mouse mastocytoma cell line, P815-HTR : Mouse mastocytoma cell line(P815-HTR)을 이용한 세로토닌 합성 효소 유전자(Tryptophan hydroxylase)의 발현 및 조절에 관한 연구

저자 : 이창욱
발행 : 춘천 : 한림대학교 대학원, 2000

Bender T P , 1996, in Current Protocols in Molecular Biology) and using a modification of published methods (Abcouwert

문장표절률: **20%**

F., Schwarz, C., and Meguid, R. A., 1999, J. Biol. Chem.). All procedures involving extraction of the nuclei were performed at 4 ℃. For in vitro transcription reactions, 3 10 7 nuclei were incubated for 30 min at 30 ℃ in transcription buffer (Greenberg.

[Copykiller] Microbial BMAA elicits mitochondrial dysfunction, innate immunity activation, and Alzheimer's disease features in cortical neurons

저자 : [Author(affiliation=CNC-Center for Neuroscience and Cell Biology, University of Coimbra, name=Diana F. Silva), Author(affiliation=CNC-Center for Neuroscience and Cell Biology, University of Coimbra, name=Emanuel Candeias), Author(affiliation=CNC-Center for Neuroscience and Cell Biology, University of Coimbra, name=A. Raquel Esteves), Author(affiliation=CNC-Center for Neuroscience and Cell Biology, University of Coimbra, name=João D. Magalhães), Author(affiliation=CNC-Center for Neuroscience and Cell Biology, University of Coimbra, name=I. Luísa Ferreira), Author(affiliation=CNC-Center for Neuroscience and Cell Biology, University of Coimbra, name=Daniela Nunes-Costa), Author(affiliation=CNC-Center for Neuroscience and Cell Biology, University of Coimbra, name=A. Cristina Rego), Author(affiliation=CNC-Center for Neuroscience and Cell Biology, University of Coimbra, name=Nuno Empadinhas), Author(affiliation=CNC-Center for Neuroscience and Cell Biology, University of Coimbra, name=Sandra M. Cardoso)]
발행 : 2020-11-08T12:16:23Z

μM oligomycin. Then, mitochondria were incubated for 30 min at 30 ℃ in the absence or in the

[Copykiller] 식품고압기술개발사업

발행 : 2014

instructions. Briefly, samples were incubated for 30 min at 30 ℃ in a precoated plate with a

문장표절률: **47%**

M) containing 250 mCi of [-32P] UTP. Following chromatin disruption with the high salt buffer, DNase treatment, and isopropyl alcohol precipitation, RNA was resuspended in 0.1% SDS, diluted with lysis buffer RTL (Qiagen), and then purified using the RNeasy total RNA extraction kit (Qiagen) and following the manufacturer's instructions. Transcripts were captured by linearized and denatured

[Copykiller] Studies on the tryptophan hydroxylase gene expression in mouse mastocytoma cell line, P815-HTR : Mouse mastocytoma cell line(P815-HTR)을 이용한 세로토닌 합성 효소 유전자(Tryptophan hydroxylase)의 발현 및 조절에 관한 연구

저자 : 이창욱
발행 : 춘천 : 한림대학교 대학원, 2000

UTP. Following chromatin disruption with high salt buffer, DNase treatment, and isopropyl alcohol precipitation, RNA was resuspended in 0.1% sodium dodecylsulfate SDS diluted with lysis buffer RTL (Qiagen), and then purified using the RNeasy™ total RNA extraction

문장표절률: **41%**

plasmids (described above for Northern blotting) were immobilized on nylon membranes at 5 g/slot. Capture membranes were blocked for 15-30 min in prehybridization solution (Greenberg, M.) at 65 ℃ and then incubated with 1 10 7 cpm/ml of purified in vitro labeled RNA diluted in hybridization solution (Greenberg, M.) for 30 hr at 65 ℃.

[Copykiller] Studies on the tryptophan hydroxylase gene expression in mouse mastocytoma cell line, P815-HTR : Mouse mastocytoma cell line(P815-HTR)을 이용한 세로토닌 합성 효소 유전자(Tryptophan hydroxylase)의 발현 및 조절에 관한 연구

저자 : 이창욱
발행 : 춘천 : 한림대학교 대학원, 2000

nylon membranes at 5 ug/slot. Capture membranes were blocked for 15-30 min in prehybridization solution at 65 ℃ and then ...... 1 x 1 □7 cpm/ml of purified in vitro labeled RNA diluted in hybridization solution for 30 h at 65

문장표절률: **42%**

After extensive washing at 65 ℃ with 1 SSC containing 0.1% SDS, the membranes were treated with 50 g/ml RNase A (Qiagen) for 30 min at room temperature and then washed at room temperature with 1 SSC containing 0.1% SDS.

[Copykiller] Studies on the tryptophan hydroxylase gene expression in mouse mastocytoma cell line, P815-HTR : Mouse mastocytoma cell line(P815-HTR)을 이용한 세로토닌 합성 효소 유전자(Tryptophan hydroxylase)의 발현 및 조절에 관한 연구

저자 : 이창욱
발행 : 춘천 : 한림대학교 대학원, 2000

were treated with 50 ug/ml RNase A (Qiagen) for 30 min at room temperature and then washed at room temperature with 1 x standard saline citrate SSC

문장표절률: **0%**

Captured transcripts were detected by exposing the membranes to x-ray film (Reflection, NEN Life Science).

문장표절률: **0%**

RNA stability assay P815-HTR cells were seeded in 6-well plates in completeDMEM for 12 hr. The cells were then rinsed twice with GLN-free medium, fed with GLN-free mediasupplemented with or without 4 mM GLN in combinations of 10 M SB203580 (Tocris), 10 g/ml actinomycin D (Sigma) or 100 M 5,6-dichloro-1-b-D-ribofuranosylbenzimidazole (DRB, Calbiochem) in.

문장표절률: **0%**

The SB203580 reagent was added from a 10 mM stock in DMSO, and actinomycin D was added from a 10 mg/ml stock in absolute ethanol.

Copyright © MUHAYU All Rights reserved.

문장표절률: 0%

The DRB was added from a 100 mM stock in DMSO. The control cultures wer etreated with the given carrier solution without the added drug.

문장표절률: 0%

At the indicated times after treatment, the cultures were harvested and total RNA was isolated as described for Northern blotting.

문장표절률: 20%

Statistical analysis Statistical analyses was via GraphPad Prism version 5 softwa re (GraphPad). The data arepresented as mean± standard error, and were com pared statistically by Student-t test (for 2 groups) and ANOVA with "Tukey's M ultiple Comparison Test" (for more than 2 groups). Significant differences were i ndicated for p<0.05.

[Copykiller] Studies on polysaccharides using as prebiotics and their effects o n human diseases = 프로바이오틱스로 이용 가능한 다당류 및 임상적 효능에 관한 연구

저자 : PHAM THI NGOC ANH
발행 : 2021

analysis followed by post hoc Tukey's multiple comparison test for more than 2 groups.

문장표절률: 0%

Results Levels of TPH1 mRNA in P815-HTR cells were increased by presence of GLN, which was blocked by a GLN antagonist The P815-HTR cells were cu ltured incomplete medium before each experiment.

문장표절률: 0%

The subconfluent cells were re-fed with fresh culture medium containing 4 mM GLN, and the levels of TPH1 mRNA were

문장표절률: 0%

determined at the indicated time points (Fig. 1A and B). Themedium exchange c aused an increase in TPH1 mRNA levels at 6 hr post treatment, mostly irrespecti ve of presence of serum (Fig.

문장표절률: 0%

1A and B). The exchange with fresh medium was thought to contain a serum-in dependent factor that might increase TPH1 mRNA levels.

문장표절률: 0%

Through several experiments, it was suspected that GLN in the medium have ca used the induction of TPH1 mRNA. Fig. 1B shows a robust, time-dependent inc rease in TPH1 mRNA levels upon presence of 4 mM GLN in themedium.

문장표절률: 0%

This effect was confirmed by treatment with the specific GLN antagonist, 6-dia zo-5-oxo-L-norleucine (DON) [25], dose-dependently blocking the increases (Fig.

문장표절률: 0%

1C). DON is structurally similar to GLN; due to its reactive diazo group, DON alkylates and inhibits GLN utilizing enzymes such glutaminase; it is used to bloc k GLN dependent pathways in the cell [25].

문장표절률: 0%

From these results, we demonstrated that exogenous GLN induces a massive in crease in TPH1 mRNA levels in P815-HTR cells (approximately 2-fold).

문장표절률: 0%

Inhibition of p38 MAPK blocks the increase in TPH1 mRNA levels by GLN We nextasked whether the observed increases in GLN-led TPH1 mRNA increases were dependent on MAP kinase or PI3K pathways.

Copyright © MUHAYU All Rights reserved.

문장표절률: 39%

The agents used were SB203580 (p38 MAPK inhibitor), PD98059 (p42/44 MAPK inhibitor) and wortmannin (PI3K inhibitor) and the treated samples were compared with the control (carrier treated) TPH1 mRNA levels [26, 27].

[Copykiller] Studies on the tryptophan hydroxylase gene expression in mouse mastocytoma cell line, P815-HTR : Mouse mastocytoma cell line(P815-HTR)을 이용한 세로토닌 합성 효소 유전자(Tryptophan hydroxylase)의 발현 및 조절에 관한 연구

저자 : 이창욱
발행 : 춘천 : 한림대학교 대학원, 2000

p38 MAPK inhibitor SB203580 As SB203580 (p38 MAPK inhibitor), PD98059 (p42/44 MAPK inhibitor) and wortmannin (PI3K inhibitor) have different intracellular targets, we

문장표절률: 0%

Subconfluent cells were fed complete cultured medium, containing 4 mM GLN, in presence of SB203580, PD98059, or wortmannin for 6 hr, and the changes in TPH1 mRNA levels were measured (Fig.

문장표절률: 0%

2). Only the p38 MAPK inhibitor, SB203580, was seen to abolish the GLN induction of TPH1 mRNA at 6 hr (Fig. 2). The P815-HTR cells were then cultured for the indicated times in fresh DMEM in absence or presence of 4 mM GLN and activation of p38 kinase was confirmed by GLN treatment (Fig.

문장표절률: 0%

3A; right panel). As p38 mitogen-activated protein kinase is also activated by bacterial lipopolysaccharide (LPS) [28], we examined whether P815-HTR cells activated in their p38 kinase post a 6 hr exposure to LPS containing medium in absence of GLN would alter their abundance of TPH1 mRNA as measured by northern blot analysis (Fig.

문장표절률: 0%

3B). The above was indeed observed with the conclusion that p38 kinase activity is essential for TPH1 mRNA induction by GLN and activation of p38 MAPK pathway can substitute for the treatment of the cells by GLN.

문장표절률: 0%

Transcription of TPH1 gene is increased by GLN The increases in the levels of TPH1 mRNA due to exogenous GLN could be from a number of mechanisms, such as augmented transcription, improved RNA stability, or both.

문장표절률: 0%

To test the effects of exogenous GLN on TPH1 gene transcription, a nuclear run-on assay was performed to determine transcription initiation rate for TPH1 gene.

문장표절률: 0%

Subconfluent P815-HTR cells were washed and incubated with medium in presence or absence of 4 mM GLN. Following a 5.5 hr incubation, the cellular nuclei were harvested.

문장표절률: 0%

Equivalent purified and in vitro [-32P] UTP labeled RNA was incubated on membranes for run-on transcription of nuclei from the GLN-containing and GLN-withheld cells.

문장표절률: 0%

The results are shown with the GAPDH housekeeping control along with cyclophilin (CPN) used as internal RNA standards (Fig.

문장표절률: 30%

4). For RNA derived from the nuclei of GLN-fed cells, the amount of radioactivity captured by the TPH1 cDNA was only slightly increased (by less than 0.3-fold).

[Copykiller] Studies on the tryptophan hydroxylase gene expression in mouse mastocytoma cell line, P815-HTR : Mouse mastocytoma cell line(P815-HTR)을 이용한 세로토닌 합성 효소 유전자(Tryptophan hydroxylase)의 발현 및 조절에 관한 연구

저자 : 이창욱
발행 : 춘천 : 한림대학교 대학원, 2000

derived from nuclei of Gin-fed cells, the amount of radioactivity captured by the TPH cDNA was slightly increased

Copyright © MUHAYU All Rights reserved.

문장표절률: **0%**

Similar results were obtained for three repeats. As the overall increases in TPH1 transcription by GLN were always by 2 to 3-fold, this suggested that GLN causes up-regulation of TPH1 mRNA levels by different means and not solely by increased transcription.

문장표절률: **0%**

TPH1 mRNA is intrinsically unstable and inhibition of p38 MAPK accelerates its decay The p38 MAPK activity in cells may modulate the half-life of TPH1 mRNA; as such, changes in the half-life of TPH1 mRNA in cells post GLN feeding were studied.

문장표절률: **0%**

Subconfluent P815-HTR cells were first fed and cultured in 4 mM GLN containing medium for approximately 14 hr, followed by 10 hr of monitoring their TPH-1 RNA levels after transferring them to GLN-free culture conditions (Fig.

문장표절률: **0%**

5). The changes in the levels of mRNA were compared with that of cyclophilin (CPN) mRNA, with normalized values at various times post monitoring of mRNA.

문장표절률: **0%**

Compared with CPN RNA message, there was a steady decline in TPH1 mRNA levels in control treated cells when they were cultured in GLN-free media, and this was similar when in presence of either of two transcription inhibitors, actinomycin D or DRB (53-85-0), blocking transcription via various means [29].

문장표절률: **0%**

This implied that themessage levels for TPH1 are intrinsically unstable and they go through a fast decay when cells run down their sources of GLN (Fig.

문장표절률: **0%**

5). Treatment of the cells with p38 MAPK inhibitor, SB203580, accelerated the decay of TPH1 mRNA, which was blunted with a global transcriptional block (SB203580 in presence of actinomycin D or SB203580 in presence of DRB).

문장표절률: **0%**

The rate of SB203580-led TPH1 mRNA decay was higher than the baseline decay, with SB203580 treatment enhancing the degradation and destabilization of TPH1 mRNA.

문장표절률: **0%**

Presence of actinomycin D or DRB treatment blunted the effect of SB203580, leading us to hypothesize that the p38 MAPK activity inhibits transcription of certain gene(s) mediating TPH1 mRNA destabilization.

출처표시 문장 문장표절률: **0%**

Discussion In this report, we describe the mechanism for accumulation of TPH1 mRNA in cultured P815-HTR mouse mastocytoma cells; it should be noted that TPH enzymatic activity in P815-HTR cells has been previously reported (Reed et al., 1995).

문장표절률: **0%**

Due to the unavailability of a TPH-positive cell line with a pineal or neuronal lineage, the P815 and P815-HTR (high transfection) lines were used for analysis of TPH1 transcription regulation and the factors affecting its RNA half-life.

Copyright © MUHAYU All Rights reserved.

문장표절률: 25%

We showed that exchange of culture medium stimulated the induction of TPH1 mRNA. This effect was independent of presence of serum and depended on presence of exogenous GLN in themedia of the cultured cells (Fig. 1A).

[Copykiller] Studies on the tryptophan hydroxylase gene expression in mouse mastocytoma cell line, P815-HTR : Mouse mastocytoma cell line(P815-HTR)을 이용한 세로토닌 합성 효소 유전자(Tryptophan hydroxylase)의 발현 및 조절에 관한 연구

저자 : 이창욱
발행 : 춘천 : 한림대학교 대학원, 2000

We shown that exchange of culture medium stimulated the induction of TPH mRNA. This effect was

문장표절률: 0%

GLN is a required nutrient for cell manufacture of purines and pyrimidines and also a usable energy source for the cell, shown for cultured mammalian cells and also many tumor cells [30] as GLN plays a key role in tumor cell metabolism, with multiple tumor types using GLN as their major respiratory fuel [31].

문장표절률: 0%

We hypothesized that the cellular GLN might be important in regulating TPH1 mRNA expression in P815-HTR cells. Consistent with this view, use of a glutamine antagonist, 6-diazo-5-oxo-L-norleucine (DON), suppresses the TPH1 mRNA induction by exogenous GLN (Fig.

문장표절률: 0%

1B, C). DON is a glutamine anti-metabolite and it blocks mitochondrial glutaminases and amidotransferases that are dependent on glutamine.

문장표절률: 0%

We found p38 MAPK activity to be increased by GLN treatment of P815-HTR cells (Fig. 2) and was essential for stability of TPH1 mRNA in those cells (Fig.

문장표절률: 0%

5). Similarly, p38 MAPK has been documented to be involved in Cox-2 mRNA stability [32]. In those reports, p38-dependent Cox-2 mRNA stabilization was via the p38 substrate MAPKAPK-2 and was probably in part due to the small heat shock protein hsp27 becoming phosphorylated.

문장표절률: 22%

The increased RNA half-life due to p38 MAPK was considered to be gene specific, as 3' UTR sequences derived from c-myc or TNF also destabilized the -globin reporter transcript but were not responsive to the p38 MAPK signaling [32].

[Copykiller] Roles of cAMP in regulation of tryptophan hydroxylase(TPH) gene expression in serotonergic CA77 cells = 세로토닌성 CA77세포주에서 cAMP에 의한 Tryptophan Hydroxylase(TPH)유전자 조절에 관한 연구

저자 : 권용준
발행 : 춘천 : 한림대학교 대학원, 2005.2

mRNA consider as sequencespecific, since 3' UTR sequences derived from c-myc or TNF a destabilize the p-globin

[Copykiller] Roles of cAMP in regulation of tryptophan hydroxylase(TPH) gene expression in serotonergic CA77 cells = 세로토닌성 CA77세포주에서 cAMP에 의한 Tryptophan Hydroxylase(TPH) 유전자 조절에 관한 연구

저자 : 權龍俊
발행 : 춘천: 翰林大學校, 2004

mRNA consider as sequencespecific, since 3' UTR sequences derived from c-myc or TNF a destabilize the p-globin

문장표절률: 0%

The p38 pathway has also been shown to regulate the half-life of reporter transcripts having GM-CSF, c-fos, IL-6, and IL-8 response elements (ARE) [33, 34].

문장표절률: 0%

In this study, we demonstrate that p38 kinase activation elicits a substantial increase in thestability of TPH1 mRNA. But the putative AREs do not exist in TPH1 3'UTR for p38-mediated stability of TPH1 message; therefore, it seems that they are distinct mechanisms other than cis-acting sequences in regulating TPH1 mRNA stability.

문장표절률: 0%

In our study, transcription of thestability of TPH1 mRNA was studied in presence or absence of actinomycin D or DRB transcription inhibitors [29].

Copyright © MUHAYU All Rights reserved.

문장표절률: **0%**

The chance of actinomycin D with DRB directly modulating the processes in TPH1 mRNA decay couldnot be excluded, as

문장표절률: **0%**

transcriptional inhibitors may affect the nuclear-to-cytoplasmic trafficking of a number of RNA binding proteins [35-37], and directly affect the stability of certain mRNA species [38].

문장표절률: **33%**

We do not believe that this was the case in our study, as the rate of actinomycin D or DRB treated samples for TPH1 mRNA decay were similar to that of their intrinsic RNA decay (Fig.

[Copykiller] Roles of cAMP in regulation of tryptophan hydroxylase(TPH) gene expression in serotonergic CA77 cells = 세로토닌성 CA77세포주에서 cAMP에 의한 Tryptophan Hydroxylase(TPH)유전자 조절에 관한 연구

저자 : 권용준
발행 : 춘천 : 한림대학교 대학원, 2005.2

We do not believe that this was the case in our study, since the rate of actinomycin

[Copykiller] Roles of cAMP in regulation of tryptophan hydroxylase(TPH) gene expression in serotonergic CA77 cells = 세로토닌성 CA77세포주에서 cAMP에 의한 Tryptophan Hydroxylase(TPH)유전자 조절에 관한 연구

저자 : 권용준
발행 : 춘천 : 한림대학교 대학원, 2005.2

We do not believe that this was the case in our study, since the rate of actinomycin

문장표절률: **0%**

5A). Another caveat in this study is that at high concentrations, SB203580 may inhibit some JNK activity [39]; however, activation of JNK by GLN was not observed (data not shown).

문장표절률: **0%**

On the role of environmental GLN in regulating the levels and activity of TPH1, we hypothesize that exogenous GLN via TPH1 physiologically affects the stores of serotonin and its metabolites [6].

문장표절률: **0%**

These may have profound effects as there are a myriad of functions in the periphery for serotonin [3]. GLN levels are also modulated in feeding, starvation, health and disease [20], and these may lead to serotonin level changes.

문장표절률: **0%**

Interestingly, as the blood-brain barrier blocks the ready traverse of serotonin into and out of the CNS, the peripheral and central serotonin compartments are thought to be functionally separate and any systemic changes of GLN may have varying effects on a given compartment of serotonin [40].

문장표절률: **0%**

Serotonin is also used in serotonylation of various proteins such as fibronectin, certain GTPase proteins, and histones [3].

문장표절률: **0%**

This also adds another layer of complexity to the physiological effects of GLN changes on cellular substrates of serotonin.

문장표절률: **0%**

All of these areas need to be addressed in animalstudies for physiological changes due to GLN that affectserotonin.

문장표절률: **0%**

Conclusion The details of environmental GLN and subsequent signaling for transcriptional increase and stabilization of TPH1 mRNA remain to be addressed and are deemed to be significant as dramatically up-regulated levels of TPH1 message represent a potential mechanism for the long observed and yet poorly understood GLN effects on various mammalian cells.

Copyright © MUHAYU All Rights reserved.

문장표절률: **0%**

Acknowledgments None. Funding This research was supported by Hallym University Research Fund, (HRF-202002-015) to Sung-Oh Huh.

문장표절률: **100%**

Compliance with ethical standards Conflict of interest All authors declare that there are no conflicts of interest.

[www.researchgate.net] (PDF) Therapeutic potential of quercetin on human breast cancer in ...

발행 : www.researchgate.net

or not-for-profit sectors. Compliance with ethical standards Conflict of interest All authors declare that there are no conflicts of interest

[link.springer.com] Anorexia in human and experimental animal models: physiological ...

발행 : link.springer.com

by The Naito Foundation, Japan. Conflict of interest All authors declare that there are no conflicts of interest, financial or otherwise. Author information

문장표절률: **56%**

Informed consent Informed consent was received from all individual participants included in the study. Author contributions H.P.

[Copykiller] Short-Term Meditation Training Fosters Mindfulness and Emotion Regulation: A Pilot Study

저자 : [Author(affiliation=Department of Brain and Behavioral Sciences, University of Pavia, Pavia, Italy, name=Teresa Fazia), Author(affiliation=Department of Brain and Behavioral Sciences, University of Pavia, Pavia, Italy, name=Francesco Bubbico), Author(affiliation=Department of Brain and Behavioral Sciences, University of Pavia, Pavia, Italy, name=Ioannis Iliakis), Author(affiliation=Department of Brain and Behavioral Sciences, University of Pavia, Pavia, Italy, name=Gerardo Salvato), Author(affiliation=Cognitive Neuropsychology Centre, ASST "Grande Ospedale Metropolitano" Niguarda, Milan, Italy, name=Gerardo Salvato), Author(affiliation=Department of Brain and Behavioral Sciences, University of Pavia, Pavia, Italy, name=Giovanni Berzuini), Author(affiliation=Istituto di Psicosintesi, Milan, Italy, name=Salvatore Bruno), Author(affiliation=Department of Brain and Behavioral Sciences, University of Pavia, Pavia, Italy, name=Luisa Bernardinelli)]

발행 : 2020-10-26T04:45:25Z

authors. Informed consent was obtained from all individual participants included in the study. AUTHOR CONTRIBUTIONS LB designed the study. TF

[Copykiller] Role of Roflumilast Combined with ESHAP Chemotherapy in Relapsed/Refractory Patients with Diffuse Large B-Cell Lymphoma Role of Roflumilast Combined with ESHAP Chemotherapy in Relapsed/Refractory Patients with Diffuse Large B-Cell Lymphoma

저자 : Do Young Kim Jehyun Nam Joo-seop Chung Sang-Woo Kim Ho-Jin Shin

발행 : 2021/04/27

and informed consent was obtained from all individual participants included in the study. Author Contributions Conceived and designed the analysis

문장표절률: **0%**

and C.W.L. carried outmost of the experiments; J.K. analyzed the data; A.S. wrote and revised a part of the manuscript; S-O. H. designed the project, wrote the manuscript, arranged the grants and led the team to accomplish it.

문장표절률: **100%**

All authors commented on previous versions of the manuscript. All authors read and approved the final manuscript.

[behavioralandbrainfunctions.biomedcentral.com] The neural correlates of mental arithmetic in adolescents: a ...

발행 : behavioralandbrainfunctions.biomedcentral.com

all authors commented on previous versions of the manuscript. All authors read and approved the final manuscript

[bmcgeriatr.biomedcentral.com] Prevalence and incidence of cognitive impairment in an elder ...

저자 : Pais, Ricardo, Ruano, Luís, Moreira, Carla, Carvalho, Ofélia P., Barros, Henrique

발행 : 2020/11/16

all authors commented on previous versions of the manuscript. All authors read and approved the final manuscript

Copyright © MUHAYU All Rights reserved.

참고문헌

References 1. Lin SH, Lee LT, Yang YK (2014) Serotonin and mental disorders: a concise review on molecular neuroimaging evidence. Clin Psychopharmacol N eurosci 12:196-202. https://doi.org/10.9758/cpn.2014.12.3.196. 2. Bellono N W, Bayrer JR, Leitch DB, Castro J, Zhang C, O'Donnell TA, et al. (2017) Entero chromaffin Cells Are Gut Chemosensors that Couple to Sensory Neural Pathwa ys. Cell 170:185-98 e16. https://doi.org/10.1016/j.cell.2017.05.034. 3. Jiang S H, Wang YH, Hu LP, Wang X, Li J, Zhang XL, et al. (2021) The physiology, p athology and potential therapeutic application of serotonylation. J Cell Sci 134. h ttps://doi.org/10.1242/jcs.257337. 4. Bader M (2019) Serotonylation: Serotoni n Signaling and Epigenetics. Front Mol Neurosci 12:288. https://doi.org/10.33 89/fnmol.2019.00288. 5. Muma NA, Mi Z (2015) Serotonylation and Transami dation of Other Monoamines. ACS Chem Neurosci 6:961-9. https://doi.org/1 0.1021/cn500329r. 6. Slominski AT, Kim TK, Kleszczynski K, Semak I, Janjetov ic Z, Sweatman T, et al. (2020) Characterization of serotonin and N-acetylserot onin systems in the humanepidermis and skin cells. J Pineal Res 68:e12626. https ://doi.org/10.1111/jpi.12626.

7. Pagan C, Delorme R, Callebert J, Goubran-Botros H, Amsellem F, Drouot X , et al. (2014) The serotonin-N-acetylserotonin-melatonin pathway as a biomar ker for autism spectrum disorders. Transl Psychiatry 4:e479. https://doi.org/10 .1038/tp.2014.120. 8. Mordhorst A, Dhandapani P, Matthes S, Mosienko V, Ro the M, Todiras M, et al. (2021) Phenylalanine hydroxylase contributes to seroto nin synthesis in mice. FASEB J 35:e21648. https://doi.org/10.1096/fj.20210036 6R. 9. Slominski A, Pisarchik A, Semak I, Sweatman T, Wortsman J, Szczesniew ski A, et al. (2002) Serotoninergic and melatoninergic systems are fully expresse d in humanskin. FASEB J 16:896-8. https://doi.org/10.1096/fj.01-0952fje. 10. Walther DJ, Bader M (2003) A unique central tryptophan hydroxylase isoform. Biochem Pharmacol 66:1673-80. https://doi.org/10.1016/s0006-2952(03)00 556-2. 11. Gentile MT, Nawa Y, Lunardi G, Florio T, Matsui H, Colucci-D'Am ato L (2012) Tryptophan hydroxylase 2 (TPH2) in a neuronal cell line: modulati on by cell differentiation and NRSF/rest activity. J Neurochem 123:963-70. http s://doi.org/10.1111/jnc.12004.

12. Grohmann M, Hammer P, Walther M, Paulmann N, Buttner A, Eisenmenger W, et al. (2010) Alternativesplicing and extensive RNA editing of human TPH2 t ranscripts. PLoS One 5:e8956. https://doi.org/10.1371/journal.pone.0008956. 13. Cote F, Schussler N, Boularand S, Peirotes A, Thevenot E, Mallet J, et al. (2 002) Involvement of NF-Y and Sp1 in basal and cAMP-stimulated transcriptio nal activation of the tryptophan hydroxylase (TPH ) gene in the pineal gland. J Neurochem 81:673-85. https://doi.org/10.1046/j.1471-4159.2002.00890.x. 1 4. Patrick RP, Ames BN (2014) Vitamin D hormone regulates serotonin synthesi s. Part 1: relevance for autism. FASEB J 28:2398-413. https://doi.org/10.1096 /fj.13-246546. 15. Chaudhary P, Guragain D, Chang JH, Kim JA (2021) TPH1 and 5-HT7 Receptor Overexpression Leading to Gemcitabine-Resistance Requi res Non-Canonical Permissive Action of EZH2 in Pancreatic Ductal Adenocarc inoma. Cancers (Basel) 13. https://doi.org/10.3390/cancers13215305. 16. Joha nsson TA, Westin G, Skogseid B (2009) Identification of Achaete-scute comple x-like 1 (ASCL1) target genes and evaluation of DKK1 and TPH1 expression in pancreatic endocrine tumours. BMC Cancer 9:321. https://doi.org/10.1186/14 71-2407-9-321.

17. Stoll J, Kozak CA, Goldman D (1990) Characterization and chromosomal mapping of a cDNA encoding tryptophan hydroxylase from a mouse mastocyt oma cell line. Genomics 7:88-96. https://doi.org/10.1016/0888-7543(90)905 22-v. 18. Sakowski SA, Geddes TJ, Thomas DM, Levi E, Hatfield JS, Kuhn DM (2006) Differential tissuedistribution of tryptophan hydroxylase isoforms 1 and 2 as revealed with monospecific antibodies. Brain Res 1085:11-8. https://doi.or g/10.1016/j.brainres.2006.02.047. 19. Yoo HC, Yu YC, Sung Y, Han JM (2020 ) Glutamine reliance in cell metabolism. Exp Mol Med 52:1496-516. https://doi. org/10.1038/s12276-020-00504-8. 20. Cruzat V, Macedo Rogero M, Noel Keane K, Curi R, Newsholme P (2018) Glutamine: Metabolism and Immune Fu nction, Supplementation and Clinical Translation. Nutrients 10. https://doi.org/1 0.3390/nu10111564. 21. Etienne TA, Cocaign-Bousquet M, Ropers D (2020) Competitive effects in bacterial mRNA decay. J Theor Biol 504:110333. https:// doi.org/10.1016/j.jtbi.2020.110333. 22. Basu S, Mallik S, Hait S, Kundu S (2021 ) Genome-scale molecular principles of mRNA half-life regulation in yeast. FEB S J 288:3428-47. https://doi.org/10.1111/febs.15670.

Copyright © MUHAYU All Rights reserved.

23. Rhoads JM, Argenzio RA, Chen W, Graves LM, Licato LL, Blikslager AT, et al. (2000) Glutamine metabolism stimulates intestinal cell MAPKs by a cAMP-inhibitable, Raf-independent mechanism. Gastroenterology 118:90-100. https://doi.org/10.1016/s0016-5085(00)70417-3. 24. Haussinger D, Schliess F, Dombrowski F, Vom Dahl S (1999) Involvement of p38MAPK in the regulation of proteolysis by liver cell hydration. Gastroenterology 116:921-35. https://doi.org/10.1016/s0016-5085(99)70076-4. 25. Thangavelu K, Chong QY, Low BC, Sivaraman J (2014) Structural basis for the active site inhibition mechanism of human kidney-type glutaminase (KGA). Sci Rep 4:3827. https://doi.org/10.1038/srep03827. 26. Kuo JR, Cheng YH, Chen YS, Chio CC, Gean PW (2013) Involvement of extracellular signal regulated kinases in traumatic brain injury-induced depression in rodents. J Neurotrauma 30:1223-31. https://doi.org/10.1089/neu.2012.2689. 27. Chin A, Svejda B, Gustafsson BI, Granlund AB, Sandvik AK, Timberlake A, et al. (2012) The role of mechanical forces and adenosine in the regulation of intestinal enterochromaffin cell serotonin secretion. Am J Physiol Gastrointest Liver Physiol 302:G397-405. https://doi.org/10.1152/ajpgi.00087.2011.

28. Dumitru CD, Ceci JD, Tsatsanis C, Kontoyiannis D, Stamatakis K, Lin JH, et al. (2000) TNF-alpha induction by LPS is regulated posttranscriptionally via a Tpl2/ERK-dependent pathway. Cell 103:1071-83. https://doi.org/10.1016/s0092-8674(00)00210-5. 29. Bensaude O (2011) Inhibiting eukaryotic transcription: Which compound to choose? How to evaluate its activity? Transcription 2:103-8. https://doi.org/10.4161/trns.2.3.16172. 30. Shanware NP, Mullen AR, DeBerardinis RJ, Abraham RT (2011) Glutamine: pleiotropic roles in tumor growth and stress resistance. J Mol Med (Berl) 89:229-36. https://doi.org/10.1007/s00109-011-0731-9. 31. Yang L, Venneti S, Nagrath D (2017) Glutaminolysis: A Hallmark of Cancer Metabolism. Annu Rev Biomed Eng 19:163-94. https://doi.org/10.1146/annurev-bioeng-071516-044546. 32. Zhao Y, Liu X, Qu Y, Wang L, Geng D, Chen W, et al. (2019) The roles of p38 MAPK --> COX2 and NF-kappaB --> COX2 signal pathways in age-related testosterone reduction. Sci Rep 9:10556. https://doi.org/10.1038/s41598-019-46794-5.

33. Salvador-Bernaldez M, Mateus SB, Del Barco Barrantes I, Arthur SC, Martinez AC, Nebreda AR, et al. (2017) p38alpha regulates cytokine-induced IFNgamma secretion via the Mnk1/eIF4E pathway in Th1 cells. Immunol Cell Biol 95:814-23. https://doi.org/10.1038/icb.2017.51. 34. Dauletbaev N, Eklove D, Mawji N, Iskandar M, Di MarcoS, Gallouzi IE, et al. (2011) Down-regulation of cytokine-induced interleukin-8 requires inhibition of p38 mitogen-activated protein kinase (MAPK) via MAPK phosphatase 1-dependent and -independent mechanisms. J Biol Chem 286:15998-6007. https://doi.org/10.1074/jbc.M110.205724. 35. Nazer E, Verdun RE, Sanchez DO (2011) Nucleolar localization of RNA binding proteins induced by actinomycin D and heat shock in Trypanosoma cruzi. PLoS One 6:e19920. https://doi.org/10.1371/journal.pone.0019920. 36. Glisovic T, Soderberg M, Christian K, Lang M, Raffalli-Mathieu F (2003) Interplay between transcriptional and post-transcriptional regulation of Cyp2a5 expression. Biochem Pharmacol 65:1653-61. https://doi.org/10.1016/s0006-2952(03)00118-7. 37. Sanchez-Hernandez N, Boireau S, Schmidt U, Munoz-Cobo JP, Hernandez-Munain C, Bertrand E, et al. (2016) The in vivo dynamics of TCERG1, a factor that couples transcriptional elongation with splicing. RNA 22:571-82. https://doi.org/10.1261/rna.052795.115. 38. Otsu K, Ito K, Kuzumaki T, Iuchi Y (2001) Differential regulation of liver-specific and ubiquitously- expressed genes in primary rat hepatocytes by the extracellular matrix. Cell Physiol Biochem 11:33-40. https://doi.org/10.1159/000047790. 39. Ridley SH, Dean JL, SarsfieldSJ, Brook M, Clark AR, Saklatvala J (1998) A p38 MAP kinase inhibitor regulates stability of interleukin-1-induced cyclooxygenase-2 mRNA. FEBS Lett 439:75-80. https://doi.org/10.1016/s0014-5793(98)01342-8. 40. Matthes S, Bader M (2018) Peripheral Serotonin Synthesis as a New Drug Target. Trends Pharmacol Sci 39:560-72. https://doi.org/10.1016/j.tips.2018.03.004.

문장표절률: **0%**

Figure legends Fig. 1 Presence of GLN elevates and anti-metabolite of GLN reduces TPH1 mRNA level irrespective of serum supplement.

문장표절률: **65%**

(A) P815-HTR cells were grown in medium containing 4 mM GLN supplemented with 10% FBS for 3-days; the cells were then rinsed and fed with serumorserum-free fresh medium.

[Copykiller] Studies on the tryptophan hydroxylase gene expression in mouse mastocytoma cell line, P815-HTR : Mouse mastocytoma cell line(P815-HTR)을 이용한 세로토닌 합성 효소 유전자(Tryptophan hydroxylase)의 발현 및 조절에 관한 연구

저자 : 이창욱
발행 : 춘천 : 한림대학교 대학원, 2000

exchange and Gin-free condition. A P815-HTR cells were grown in medium containing 4 mM Gin and serum FBS 10%) for 3-days, and then rinsed and fed with serumorserum-free fresh medium.

문장표절률: **0%**

(B) After being rinsed, the cells were incubated in GLN-free medium for the indicated times and their total RNA was isolated.

Copyright © MUHAYU All Rights reserved. 

문장표절률: 56%

Equal amounts of total RNA were electrophoresed at 5 g/lane, and Northern blotting was performed as described under in the methods section utilizing DIG-labeling probes for specific genes as indicated.

[Copykiller] Studies on the tryptophan hydroxylase gene expression in mouse mastocytoma cell line, P815-HTR : Mouse mastocytoma cell line(P815-HTR)을 이용한 세로토닌 합성 효소 유전자(Tryptophan hydroxylase)의 발현 및 조절에 관한 연구

저자 : 이창욱
발행 : 춘천 : 한림대학교 대학원, 2000

RNA were electrophoresed (5 ug/lane and Northern blotting was performed as described under "Experimental Procedures," utilizing DIG-labeling probes for specific genes as indicated. Cyclophilin(CPN) was used as

문장표절률: 20%

Cyclophilin (CPN) was used as internal RNA standards. Histogram quantitation of the blot is shown. (C) Subconfluent P815-HTR cells were cultured in 4 mM GLN containing media and exposed to 5- or 10- mM DON (6-diazo-5-oxo-L-norleucine) for 6 hr.

[Copykiller] 이광자 흡수 벤조쿠마린 형광체 및 감마-글루타밀 전이효소 프로브의 개발

저자 : 노예진
발행 : 2020

cells pre-incubated with 10 mM DON (6-diazo-5-oxo-L-norleucine, 1.0 h) and the

[Copykiller] 이광자 흡수 벤조쿠마린 형광체 및 감마-글루타밀 전이효소 프로브의 개발

저자 : 노예진
발행 : 2020

cells pre-incubated with 10 mM DON (6-diazo-5-oxo-L-norleucine, 1.0 h) and the

문장표절률: 59%

Total RNA was extracted and separated, then examined by Northern blot analysis. The levels of CPN were used as the internal control.

[Copykiller] Studies on the tryptophan hydroxylase gene expression in mouse mastocytoma cell line, P815-HTR : Mouse mastocytoma cell line(P815-HTR)을 이용한 세로토닌 합성 효소 유전자(Tryptophan hydroxylase)의 발현 및 조절에 관한 연구

저자 : 이창욱
발행 : 춘천 : 한림대학교 대학원, 2000

Total RNA was extracted and separated, then examined by Northern blot analysis. The level of CPN was used

문장표절률: 90%

All histograms presented as mean ± SEM; t-test: * p ＜ 0.05, ** p ＜ 0.01, *** p ＜ 0.001, **** p ＜ 0.0001.

[www.nature.com] ShadowR: a novel chromoprotein with reduced non-specific binding ...

발행 : www.nature.com

presented as mean ± SEM t test, *P ＜ 0.05, **P ＜ 0.01, ***P ＜ 0.001, N.S. = not significant). (d ...... are indicated. The data are presented as mean ± SEM t test, *P ＜ 0.05, **P ＜ 0.01, ***P ＜ 0.001

[pubmed.ncbi.nlm.nih.gov] Time-restricted feeding is a preventative and therapeutic ...

저자 : Chaix A;Zarrinpar A;Miu P;Panda S;
발행 : 12/02/2014

iv) n=6. Data are presented as mean ± SEM t-test p ＜ 0.05, ** p ＜ 0.01, *** p ＜ 0.001. Figure 4. Time-restricted feeding

문장표절률: 0%

Fig. 2 Effects of signaling pathway inhibitors on the induction of TPH1 RNA message. P815-HTR cells were growninGLN containing medium.

문장표절률: 36%

As indicated above, the cells were then incubated with the same fresh medium or GLN-free medium in presence of various pathway inhibitors: SB203580 (p38 MAPK inhibitor), PD98059 (p42/44 MAPK inhibitor) and wortmannin (PI3K inhibitor).

[Copykiller] Studies on the tryptophan hydroxylase gene expression in mouse mastocytoma cell line, P815-HTR : Mouse mastocytoma cell line(P815-HTR)을 이용한 세로토닌 합성 효소 유전자(Tryptophan hydroxylase)의 발현 및 조절에 관한 연구

저자 : 이창욱
발행 : 춘천 : 한림대학교 대학원, 2000

p38 MAPK inhibitor SB203580 As SB203580 (p38 MAPK inhibitor), PD98059 (p42/44 MAPK inhibitor) and wortmannin (PI3K inhibitor) have different intracellular targets, we

문장표절률: 35%

After 6 hr, the cells were harvested for measurement of TPH1 mRNA abundance. Total RNA was isolated from the treated cells, equal amounts of total RNA were electrophoresed (5 μg/lane), and Northern blotting was performed as described in the methods section, utilizing DIG-labeling probes for specific genes as indicated.

[Copykiller] Studies on the tryptophan hydroxylase gene expression in mouse mastocytoma cell line, P815-HTR : Mouse mastocytoma cell line(P815-HTR)을 이용한 세로토닌 합성 효소 유전자(Tryptophan hydroxylase)의 발현 및 조절에 관한 연구

저자 : 이창욱
발행 : 춘천 : 한림대학교 대학원, 2000

Total RNA was isolated from cells, equal amounts of total RNA were electrophoresed (5 ug/lane), and Northern blotting ...... described under "Experimental Procedures, utilizing DIG-labeling probes for specific genes as indicated. Cyclophilin(CPN) was used as

Copyright © MUHAYU All Rights reserved.

문장표절률: **63%**

Cyclophilin (CPN) was used as the internal RNA control. All histograms presented as mean ± SEM; t-test: * p < 0.05, ** p < 0.01, *** p < 0.001, **** p < 0.0001.

[www.nature.com] ShadowR: a novel chromoprotein with reduced non-specific binding ...

발행 : www.nature.com

are indicated. The data are presented as mean ± SEM t test, *P < 0.05, **P < 0.01, ***P < 0.001, N.S. = not significant). (d ...... are indicated. The data are presented as mean ± SEM t test, *P < 0.05, **P < 0.01, ***P < 0.001, N.S. = not significant).

[pubmed.ncbi.nlm.nih.gov] Time-restricted feeding is a preventative and therapeutic ...

저자 : Chaix A;Zarrinpar A;Miu P;Panda S;
발행 : 12/02/2014

iv) n=6. Data are presented as mean ± SEM t-test p < 0.05, ** p < 0.01, *** p < 0.001. Figure 4. Time-restricted feeding

문장표절률: **0%**

Fig. 3 Western blot analysis for confirming that p38 kinase is involved in TPH1 mRNA induction by environmental GLN. (A) P815-HTR cells were growninGLN containing medium.

문장표절률: **0%**

They were then fed the same fresh medium or GLN-free medium. At indicated time points, the cells were harvested for measurement of activated MAPKs.

문장표절률: **0%**

Following SDS-polyacrylamide slab gel electrophoresis, the resolved proteins were transferred onto PVDF membrane and blotted by Western probing.

문장표절률: **0%**

The probed proteins were visualized as described in the methods section. (B) After being rinsed, the cells were stimulated with bacterial lipopolysaccharide (LPS) at 1 g/ml, a potent activator of p38 MAPK.

문장표절률: **0%**

After 6 hr, the cells were harvested for measurement of TPH1 mRNA abundance. Total RNA was isolated from the cells, and equal amounts of total RNA were electrophoresed at 5 g/lane.

문장표절률: **54%**

Northern probing was detailed in the methods section utilizing DIG-labeling probes for specific genes as indicated. Cyclophilin (CPN) was used as the internal RNA control.

[Copykiller] Studies on the tryptophan hydroxylase gene expression in mouse mastocytoma cell line, P815-HTR : Mouse mastocytoma cell line(P815-HTR)을 이용한 세로토닌 합성 효소 유전자(Tryptophan hydroxylase)의 발현 및 조절에 관한 연구

저자 : 이창욱
발행 : 춘천 : 한림대학교 대학원, 2000

under "Experimental Procedures," utilizing DIG-labeling probes for specific genes as indicated. Cyclophilin (CPN) was used as internal RNA standards.

문장표절률: **90%**

All histograms presented as mean ± SEM; t-test: * p < 0.05, ** p < 0.01, *** p < 0.001, **** p < 0.0001.

[www.nature.com] ShadowR: a novel chromoprotein with reduced non-specific binding ...

발행 : www.nature.com

presented as mean ± SEM t test, *P < 0.05, **P < 0.01, ***P < 0.001, N.S. = not significant). (d ...... are indicated. The data are presented as mean ± SEM t test, *P < 0.05, **P < 0.01, ***P < 0.001

[pubmed.ncbi.nlm.nih.gov] Time-restricted feeding is a preventative and therapeutic ...

저자 : Chaix A;Zarrinpar A;Miu P;Panda S;
발행 : 12/02/2014

iv) n=6. Data are presented as mean ± SEM t-test p < 0.05, ** p < 0.01, *** p < 0.001. Figure 4. Time-restricted feeding

문장표절률: **0%**

Fig. 4 Effect of environmental GLN on TPH1 transcription rates. Subconfluent P815-HTR were cultured in medium having 4 mM GLN.

Copyright © MUHAYU All Rights reserved.

문장표절률: **0%**

The cells were then washed and incubated with medium either with or without 4 mM GLN. At 6 hr post feeding, the cells were harvested, and their nuclei were is olated and cryopreserved.

문장표절률: **0%**

In a nuclear run-on assay, transcription from 30 million nuclei were allowed to proceed for 30 min at 30 C with radio-labeled UTP.

문장표절률: **0%**

Membrane capture was via binding with 5 g each of linearized/denatured plasmi ds (CPN, GAPDH, and TPH1). Two identical capture membranes were hybridiz ed for 48 hr with equal activities of 1.4 107 cpm of RNA from cells with/withou t GLN; this was proceeded by washing and treatment with RNase and exposure to film for autoradiography, as described in the methods section.

문장표절률: **0%**

Fig. 5 Effect of p38 MAPK inhibitor, SB203580, on TPH1 mRNA decay rates. ( A) Cells were fed with 4 mM GLN for overnight and then their culture was switc hed to a GLN-free one.

문장표절률: **0%**

They were also incubated with transcriptional inhibitors (actinomycin D 10 g/ml or DRD 100 M) or the carrier control, in a combination with 10 μM SB203580 as indicated in the figure.

문장표절률: **0%**

At the various times post treatment, the cells were harvested for measurement of TPH1 mRNA levels. Total RNA was isolated from the cells, and equal amounts of total RNA were electrophoresed (5 μg/lane), followed by Northern blotting o f the samples.

문장표절률: **0%**

Relative mRNA levels were calculated and plotted by comparison with CPN-nor malized at time 0, defined as the time point where the cells had been transferred t o GLN-free conditions.

Copyright © MUHAYU All Rights reserved.
